# Leveraging large-scale biobanks for therapeutic target discovery

**DOI:** 10.1016/j.xhgg.2025.100556

**Published:** 2025-12-09

**Authors:** Brian R. Ferolito, Hesam Dashti, Claudia Giambartolomei, Gina M. Peloso, Daniel J. Golden, Kai Gravel-Pucillo, Danielle Rasooly, Andrea R.V.R. Horimoto, Rachael Matty, Liam Gaziano, Yi Liu, Ines A. Smit, Barbara Zdrazil, Yakov Tsepilov, Lauren Costa, Nicole Kosik, Jennifer E. Huffman, Gian Gaetano Tartaglia, Giorgio Bini, Gabriele Proietti, Harris Ioannidis, Mohd A. Karim, Fiona Hunter, Gibran Hemani, Adam S. Butterworth, Emanuele Di Angelantonio, Claudia Langenberg, Maya Ghoussaini, Andrew R. Leach, Katherine P. Liao, Scott Damrauer, Luis E. Selva, Stacey Whitbourne, Philip S. Tsao, Jennifer Moser, Tom Gaunt, Tianxi Cai, John C. Whittaker, Million Veteran Program, Juan P. Casas, Sumitra Muralidhar, J. Michael Gaziano, Kelly Cho, Alexandre C. Pereira

**Affiliations:** 1 Million Veteran Program (MVP) Coordinating Center, Veterans Affairs Healthcare System, 2 Avenue de Lafayette, Boston, MA 02111, USA; 2 Division of Aging, Brigham and Women’s Hospital and Harvard Medical School, 75 Francis Street, Boston, MA 02115, USA; 3 Broad Institute of MIT and Harvard, 415 Main Street, Cambridge, MA 02142, USA; 4 Health Data Science Centre, Human Technopole, V.le Rita Levi-Montalcini, 1, 20157 Milan, Italy; 5 Centre for Human Technologies (CHT), Insituto Italiano di Tecnologia, Via Enrico Melen 83, Building B, 7th Floor, 16152 Genova, Italy; 6 Department of Biostatistics, Boston University School of Public Health, Crosstown Center, 801 Massachusetts Avenue, Boston, MA 02115, USA; 7 BHF Cardiovascular Epidemiology Unit, University of Cambridge, Cambridge CB2 0BB, UK; 8 MRC Integrative Epidemiology Unit, Bristol Medical School, Bristol BS8 1QU, UK; 9 European Molecular Biology Laboratory, European Bioinformatics Institute, Hinxton CB10 1SD, UK; 10 Open Targets, Hinxton CB10 1SD, UK; 11 VA Palo Alto Healthcare System, Palo Alto, CA 94304, USA; 12 Department of Medicine, Harvard Medical School, Boston, MA 02115, USA; 13 ICREA - Institució Catalana de Recerca i Estudis Avançats, Passeig Llúis Companys 23, 08010 Barcelona, Spain; 14 Università degli Studi di Genova, Via Balbi 5, 16126 Genova, Italy; 15 Genomic Discovery, Variant Bio, Seattle, WA 98109, USA; 16 NIHR Blood and Transplant Research Unit in Donor Health and Behaviour, University of Cambridge, Cambridge CB2 0BB, UK; 17 Victor Phillip Dahdaleh Heart and Lung Research Institute, University of Cambridge, Cambridge CB2 0BB, UK; 18 British Heart Foundation Centre of Research Excellence, School of Clinical Medicine, Addenbrooke’s Hospital, University of Cambridge, Cambridge CB2 0BB, UK; 19 Health Data Research UK Cambridge, Wellcome Genome Campus, and University of Cambridge, Cambridge CB2 0BB, UK; 20 Precision Healthcare University Research Institute, Queen Mary University of London, London, UK; 21 Computational Medicine, Berlin Institute of Health at Charité – Universitätsmedizin, Berlin, Germany; 22 MRC Epidemiology Unit, University of Cambridge, Cambridge CB2 0SR, UK; 23 Regeneron Genetics Center, Regeneron Pharmaceuticals, Tarrytown, NY 10591, USA; 24 Medicine, Rheumatology, VA Boston Healthcare System, 150 S. Huntington, Boston, MA 02130, USA; 25 Division of Rheumatology, Inflammation, and Immunity, Brigham and Women’s Hospital and Harvard Medical School, 75 Francis Street, Boston, MA 02115, USA; 26 Department of Biomedical Informatics, Harvard Medical School, Boston, MA 02115, USA; 27 Corporal Michael Crescenz VA Medical Center, Philadelphia, PA 19104, USA; 28 Department of Genetics, University of Pennsylvania, Perelman School of Medicine, Philadelphia, PA 19104, USA; 29 Department of Surgery, University of Pennsylvania, Perelman School of Medicine, Philadelphia, PA 19104, USA; 30 Cardiovascular Institute, University of Pennsylvania, Perelman School of Medicine, Philadelphia, PA 19104, USA; 31 Radiology, Boston University, Chobanian and Avedisian School of Medicine, 72 East Concord Street, Boston, MA 02118, USA; 32 Palo Alto Veterans Institute for Research (PAVIR), VA Palo Alto Healthcare System, Palo Alto, CA 94304, USA; 33 Department of Medicine, Stanford University, Palo Alto, CA 94304, USA; 34 Office of Research and Development, Department of Veterans Affairs, Washington, DC 20420, USA; 35 MRC Biostatistics Unit, University of Cambridge, Cambridge CB2 0SR, UK; 36 Biomarker Development/Translational Medicine, Novartis Institutes for Biomedical Research, 250 Massachusetts Avenue, Cambridge, MA 02139, USA

**Keywords:** Mendelian randomization, genomics, target identification

## Abstract

Large biobanks, including the Million Veteran Program (MVP), the UK Biobank, and FinnGen, provide genetic association results for more than 1 million individuals for hundreds of phenotypes. To select targets for pharmaceutical development, as well as to improve the understanding of existing targets, we harmonized these studies and performed two-sample Mendelian randomization (MR) on 2,003 phenotypes using genetic variants associated with gene expression (derived from GTEx and eQTLGen) and plasma protein levels (derived from ARIC, Fenland, and deCODE) as proxies of target modulation. We found 69,669 gene-trait pairs with evidence (*p* ≤ 1.6 × 10^−9^) for causal effects. From the selected gene-trait pairs, we observed 6,447 genes with strong causal evidence for at least one of 2,003 investigated traits. As expected, being identified as a gene-trait pair in our approach was significantly associated with higher odds of being an approved drug target and indication. We were able to rediscover 9% of approved drug targets in ChEMBL 34. Moreover, identified gene-traits were significantly associated with higher odds of being previously described as a gene-trait pair in OMIM, ClinVar, mouse knockout data, and rare variant burden studies. To enhance the translational potential of the resource, we developed a predictive ranking model trained using approved drug targets described in ChEMBL 34 as well as several different biological annotations. This model was able to accurately predict the odds of a particular significant MR result being developed into an approved drug and its clinical indication (precision-recall area under the receiver operating characteristic curve 0.79). We make our results publicly available in CIPHER.

## Teaser

Using two-sample Mendelian randomization with eQTL and pQTL instruments, the study harmonizes genome-wide association study data from >1 million participants across 2,003 traits and uncovers 69,669 significant gene-trait links involving 6,447 genes, capturing 9% of approved drug targets. A follow-on machine learning model ranks each link’s therapeutic promise with a precision-recall area under the receiver operating characteristic curve of 0.79.

## Introduction

The use of genetic association data for both common and rare genetic variation has been shown to help guide target identification for drug development programs. In recent years, the availability of large-scale genetic resources such as the Million Veteran Program^1^ (MVP), the UK Biobank,^2^^,^^3^ and FinnGen^4^ have made it possible to associate millions of genetic variants to thousands of human phenotypes (or traits), including molecular phenotypes such as transcriptomics and proteomics.^5^^,^^6^^,^^7^ These efforts have had a substantial impact on our understanding of the mechanistic determinants of complex human traits, our capacity to predict medical conditions, and our ability to identify drug targets that modulate the causes of these disorders.^8^^,^^9^

Despite these advances, there are substantial hurdles to realizing the full value of these resources. For example, the identification of novel molecular targets for therapies through genome-wide association studies (GWASs) presupposes the understanding of the specific mechanism corresponding to an identified genetic association. However, genetic associations described through GWASs cannot provide more than an association estimate as a proxy to causality. Also, the lack of integration with additional genetic information maps (e.g., expression [eQTL] or protein quantitative trait locus [pQTL] data) makes GWAS results incapable of predicting the mechanism of action (MoA) of a particular gene to a phenotype, information that is fundamental for the development of a therapeutic program. To overcome some of these limitations, one would ideally need to use orthogonal sources of support, such as causal evidence from Mendelian randomization (MR), associations with rare protein-coding variants, known causal associations between genes and Mendelian disorders, insights from the human protein-protein interactome, and animal genetics, all of which are exposed to different biases. In recent years, several groups have attempted such strategies, usually focusing on a single trait or a few traits.^10^

We used causal inference methods^11^^,^^12^ associated with a large list of genetic instruments to address some of these shortcomings.^13^ In addition, the use of publicly available genetic association catalogs provides a resource for the identification and validation of novel targets.^14^ Finally, we propose that the integration of these results provides an emerging property of this approach by highlighting common mechanisms between medical entities previously considered distinct.

We meta-analyzed GWAS data from the MVP,^15^ the UK Biobank (UKBB), and FinnGen covering more than 1 million individuals and 2,003 harmonized traits, a considerable improvement in sample size and number of phenotypes compared with similar previous attempts. Then, we leveraged this resource with three GWAS-pQTLs using SOMAscan version 4 (5,000 proteins) and two GWAS-eQTL resources to conduct a genome-wide MR-phenome-wide association study (PheWAS). These resources were then combined with orthogonal sources to generate a machine learning (ML) model to quantify the odds of success of a potential target on a given disease using historical data on approved drugs.

We applied our ML model to 69,000 gene-trait associations to prioritize novel drug targets, identify indication expansion for investigational drugs, and drug repurposing opportunities for licensed drugs with higher odds of success. We believe the resources made available by this work can have broad use in drug discovery programs.

## Material and methods

### Phenotypes

The comprehensive Million Veteran Program^1^ Genome-wide Association Study × Phenome-wide Association Study (gwPheWAS) produced GWAS summary statistics for a large number of phenotypes. These phenotypes largely fit into four major categories. The first of these are clinical outcomes in the electronic health record (EHR), which are represented by phecodes. Also included were lab values, vital status measurements, and questionnaire data. There were 2,003 phenotypes available for analysis (Table S1).

### GWAS in MVP

GWASs were conducted in MVP participants using a mixed-model approach in SAIGE^16^ adjusting for age, sex, and first 10 principal components as implemented as part of the MVP gwPheWAS project. For EHR-based values, cases and controls were classified by phecodes, which comprise a high-throughput phenotyping tool based on International Classification of Diseases (ICD) codes.^17^ Individuals needed at least two phecode-mapped ICD codes to be phenotyped. Briefly, quantitative traits were inverse normal transformed to satisfy the normality assumptions before adjusting for covariates and outliers falling six standard deviations from the mean were excluded. The lab data come from clinician-adjudicated clinical labs, with all values falling within specific ranges. For each lab type, the minimum, mean, and maximum values were taken with both raw values and inverse normalized values used. Results were filtered to remove variants with poor imputation quality (*R*^2^ < 0.3) or that were very rare (minor allele frequency [MAF] <0.01% or minor allele count >20). Race and ethnicity were stratified using HARE,^18^ a supervised learning algorithm that uses both self-identified race and ethnicity, as well as genetically inferred ancestry. MVP received ethical/study protocol approval from the VA Central Institutional Review Board, and written informed consent was obtained for all participants. Detailed methods can be found in the work of Verma et al.^15^

### Harmonization of MVP, UKBB, and FinnGen for meta-analysis

To harmonize phenotypes between MVP, the Pan-UKBB,^2^ and FinnGen^4^ (version 10), traits were mapped using codes provided by the biobanks. For phenotypes that are capable of being classified by clinical diagnosis codes, MVP used entirely phecodes (1,171), while the UK Biobank used phecodes (1,327) as well as ICD10 codes (915) to label their phenotypes. After restricting studies in the UKBB to those with European ancestry in their list of populations, all possible direct phecode-to-phecode (1,013) matches were made between the biobanks (Table S1). The sample size for each phenotype from each biobank is also available in Table S1. UKBB traits with an ICD10 code were then mapped to phecodes using a conversion table,^19^ and if the derived phecode had not already been mapped to MVP in the previous step, then a match was made if possible (*n* = 10). For MVP phecodes that remained unmapped to UKBB, studies with case counts higher than 4,000 were reviewed, and a decision was made on a case-by-case basis to map manually (*n* = 53) by a clinician before aligning with FinnGen. For all unmapped MVP phecodes with case counts lower than 4,000, MVP-only data was used for analysis. To map MVP and UKBB phenotypes to FinnGen, we manually mapped all available R10 FinnGen phenotypes to those previously available from MVP or UKBB. All matches were double checked through clinical adjudication and comparison statistics using the number of cases per resource for each phenotype to identify significant outliers from this mapping strategy (see Appendix S1). Situations where a significant deviation from the overall relationship between MVP/UKBB/FinnGen case counts was observed were analyzed on a one-by-one case and a final harmonization decision was made. This led FinnGen to being mapped only with MVP (32 instances), only with UKBB (174 instances), and with both MVP and UKBB (501 instances).

### Meta-analysis between MVP, UKBB, and FinnGen

Pan-UKBB GWAS physical positions were converted from genome build GRCh37 to GRCh38 using LiftOver.^20^ Using METAL,^21^ we performed fixed effects inverse-variance weighted meta-analysis of MVP European results, UKBB European results, and FinnGen and obtained estimates of heterogeneity (option in METAL: ANALYZE HETEROGENEITY). If mapping was not possible between MVP, UKBB, or FinnGen, then only MVP results or select UKBB phenotypes were retained for further analyses. For very small *p* values in MVP that were set to zero, we set to the lowest possible decimal place allowed in Python using sys.float_info.min() (2.2250738585072014e−308). To test for inflation of *p* values following meta-analysis, we calculated the lambda values for all meta-analysis results. Any phenotype having a lambda value >1.15 (*n* = 289) was rerun, including the genomic control parameter in METAL. Phenotypes that were not meta-analyzed were also tested for inflated *p* values. We found 81 phenotypes that needed correction, the vast majority being labs (75 studies). Due to highly inflated *p* values, we removed eight height-based phenotypes from consideration. Lambda values can be found in Table S2.

### Instruments for MR

We have used five different sources of genetic instruments in the present work. Each source was analyzed independently—that is, no meta-analysis or joint analysis among sources was conducted. MR results from different sources were then analyzed using the rule-based system provided (for more information, please refer to subsection "On the use of different genetic instrument sources for MR analysis"). Below we describe the instrument selection criteria for each source. For each QTL source, all multi-SNP instruments for a given gene comprised independent genetic variants. Finally, an overall descriptive analysis of used genetic instruments can be found in Appendix S2.

### GTEx version 8

Independent *cis*-eQTLs were identified per gene by performing up to five stepwise conditional analyses in regions ±1 Mb from the transcription start site (TSS) of each gene using Genotype-Tissue Expression (GTEx) version 8^22^ individual-level data, additionally adjusting for the peak variant if there exists an association reaching a *p* value of 1e−4. The primary signal is unconditional. A total of 14,752 GTEx GENCODE version 26 genes were considered for the next steps (https://storage.googleapis.com/gtex_analysis_v8/reference/gencode.v26.GRCh38.genes.gtf).

To identify the independent signals, we considered primary and conditional associations passing a *p* value < 5e−8. We then extracted estimates of effect size and standard errors (SEs) from the unconditional association to use in the next steps of MR. This approach was taken for each available GTEx tissue. We used 120,303 markers instrumenting 24,116 genes.

### eQTLGen

Summary statistics files were downloaded from eQTLGen^23^ (https://eqtlgen.org/cis-eqtls.html). Since only *Z* scores and *p* values, but not the betas, are listed, we have computed the beta and SE from the following formulas—Beta = z/√(2p(1− p) (n + z^2^)) and SE = 1/√(2p(1− p) (n + z^2^))—after downloading the MAF file provided here: https://molgenis26.gcc.rug.nl/downloads/eqtlgen/cis-eqtl/2018-07-18_SNP_AF_for_AlleleB_combined_allele_counts_and_MAF_pos_added.txt.gz.

The summary statistics reported SNP-gene associations (<1 Mb from the center of the gene and tested in at least 2 cohorts) across 19,250 genes (17,114 in common between GTEx and eQTLGen). We defined instruments using the smallest *p* value per gene.

We used liftOver to convert SNPs from GRCh37 to GRCh38 genome build. There were 558 SNPs in GRCh37 that could not be converted and were dropped from our analysis. We used 10,669 markers instrumenting 11,175 genes.

### deCODE

We downloaded the published GWAS of SOMAscan version 4 in 35,000 individuals of European ancestry for 4,907 aptamers from deCODE^24^ (https://www.decode.com/summarydata/). We subset Table S2 from the downloaded document to only *cis*-pQTLs and removed duplicates by chromosome and position, resulting in 5,662 instruments across 1,663 genes encoding a protein (1,674 proteins and 1,703 SeqIDs). We used 4,775 markers instrumenting 1,624 genes.

### Fenland

We obtained pQTLs directly from the Fenland study,^25^ a genome-proteome-wide association study among 10,708 participants of European-descent conducted using 10.2 million genetic variants and plasma abundances of 4,775 distinct protein targets (proteins targeted by at least 1 aptamer) measured using the SOMAscan version 4 assay on 4,979 aptamers (4,775 unique protein targets). We used Table S2 reporting significant genetic variant pQTLs defined as passing a Bonferroni threshold of *p* < 1.004e−11 and secondary signals using approximate conditional analysis for each genomic region identified by distance-based clumping of association statistics. A total of 2,900 *cis*-pQTLs across 1,557 genes (mean = 1.9, minimum = 1, maximum = 14) covering an equal number of proteins from the Fenland study were used as proposed instruments. The unconditional summary statistics from this study are available in an open resource platform (www.omicscience.org). We used 2,881 markers instrumenting 1,510 genes.

### ARIC

We downloaded the published *cis*-pQTL GWAS from the Atherosclerosis Risk in Communities (ARIC) study^26^ (http://nilanjanchatterjeelab.org/pwas/), which contains SOMAscan version 4 on 4,657 plasma proteins measured in 7,213 European American individuals. The ARIC study is a prospective study conducted initially from 1987 to 1989 in four communities across the United States: Washington County, Maryland; suburbs of Minneapolis, Minnesota; Forsyth County, North Carolina; and Jackson, Mississippi. Blood samples for plasma protein data were collected from participants in the third visit in 1993–1995. SOMAmers that mapped to multiple gene targets or without a position record in the BioMart database for the target protein-coding gene or without any SNPs in the *cis*-region were excluded from further analysis. The *cis*-region is defined as ±500 kb of the TSS of the target protein-coding gene in the *cis*-pQTL analysis. Genotyping of ARIC samples was performed on the Affymetrix 6.0 DNA microarray and imputed to the TOPMed reference panel (Freeze 5b). SNPs with an MAF <1%, imputation quality (*R*^2^) <0.8, call rates <90%, or Hardy-Weinberg equilibrium *p* values <10^−6^ were removed from further analysis. A total of 2,004 significant SOMAmers were therefore identified in the original study that had at least one *cis*-pQTL (false discovery rate <5%) near the gene of the putative protein. We used unconditional estimates from this list of 2,004 *cis*-pQTLs from the original study. We used 1,612 markers instrumenting 1,594 genes.

### MR

Two-sample MR of each of 16,915 protein-coding genes were performed against all phenotypes using instruments from the 5 sources of eQTLs and pQTLs. To identify which genes were protein coding, we selected Gene type = = “protein_coding” in Ensembl Genes 108. The datasets used for instruments provided summary statistics for the unconditional primary association. For each of the datasets described above, we used the instruments identified by the authors. We extracted the corresponding effect size and SEs from the unconditional association to use in MR. To determine the correct ordering of alleles between the datasets, we utilized the harmonise_data() function from the TwoSampleMR^27^ package in R.^28^ We used the Wald ratio for instruments with one genetic variant and inverse variance weighted MR for instruments with multiple genetic variants. We additionally performed MR-Egger for proteins/expression with three or more instruments to be used as a sensitivity analysis. We tested for heterogeneity across variant-level MR estimates, using the Cochrane Q method (mr_heterogeneity option in the TwoSampleMR package) and the MR-Egger intercept.

We define genes with significant MR as genes with *p* value ≤1.6e−9 for any MR test. This value was obtained by dividing 0.05 by the number of unique gene-trait pairs tested in our study (31,525,236 unique gene-traits). If a gene was significant in multiple QTL sources, then we required the directionality of the betas to be concordant. If a gene passed in both eQTL and pQTL sources, then we considered only the pQTL.

### On the use of different genetic instrument sources for MR analysis

As detailed, we have used two different sources for eQTL instruments (GTEx and eQTLGen) and three different sources for pQTL instruments (ARIC, deCODE, and Fenland). Because prior studies have shown discrepancies between pQTLs and eQTLs, and because the specific methods of derivation are not exactly the same for each study, we have devised a set of inclusion rules of the genome-wide significant MR results that were further characterized and annotated. This arbitrary set of criteria was designed with the intent to maximize the use of the available eQTL and pQTL information in our used sources while restricting the downstream analyses to only include results that were concordant among, but not necessarily between, the different source types. We did not meta-analyze or combine different sources to create multi-SNP instruments for a gene. As such, for each gene and phenotype, MR analysis was conducted for each source independently (for GTEx, we conducted this analysis within each represented tissue). As a result, multiple MR estimates are available for each tested gene-trait. To select a particular gene-trait for downstream analysis, we only selected significant gene-traits with results where the predicted directionality of effect from MR was concordant. To be more specific, if a gene-trait was only seen as significant because of one simple instrument source, then it was accepted. If MR was significant in more than one source, then we applied the following rules: (1) for gene pairs that were significant for both eQTL and pQTL sources, we only check concordance of directionality among pQTL sources (acknowledging that eQTL and pQTL genetic proxies might be discordant due to the effects of post-transcriptional regulation); (2) the signal of the MR beta estimate had to be concordant among all significant MR results for the tested gene pair (including all different tissues from GTEx in case more than one was significant); and (3) all discordant gene-traits were excluded from further consideration.

It is thus important to consider that all derived causal effect estimates are assumed to be associated with the specific molecular mediator being instrumented—that is, the transcriptional level of a gene or the expression level of a protein in whole blood, depending on the instrument used. However, there are well-described situations where gene expression is regulated by the same genetic variant, but differently and in opposite directions at different tissues, or when RNA transcript and protein expression are regulated by the same genetic variant but in opposite directions. Conservatively, we required concordant results from all significant MR results from all different GTEx tissues, reducing the possibility of misspecification due to the former described scenario. By considering only pQTL results when these were available, we have not excluded gene-traits that could have opposite results at the eQTL and pQTL levels. A direct comparison of the correlations between each pairwise combination of significant MR beta results can be seen in Figure S1. The number of selected and excluded gene-traits on each of the steps of the selection process is illustrated in Figure S2.

### Assessment of instruments: PVE and F-statistic calculations

We compute two key parameters from the first-stage regression of the exposure phenotype on the genetic variant: the proportion of variance explained (PVE) and the F-statistic. These parameters are indicative of the power and strength of the instrumental variables (IVs) used in the study. The PVE^29^ by a given SNP can be expressed as a function of the effect size estimate (*β*), *MAF*, and standard error of effect size (*se*(*β*)) for the genetic variant, and the sample size (*n*):

$$PVE=\frac{2\beta^{2}MAF\left(1-MAF\right)}{2\beta^{2}MAF\left(1-MAF\right)+{\left(se\left(\beta \right)\right)}^{2}2nMAF\left(1-MAF\right)}$$

To capture the “strength” of the IV or set of IVs, we computed the F-statistic from the first-stage regression of the exposure phenotype on the genetic variant.^30^ The F-statistic can be expressed as a function of the *PVE*, the sample size (*n*), and the number of IVs (*k*):

$$F=\frac{PVE\left(n-1-k\right)}{\left(1-PVE\right)k}$$

We used a threshold of F < 10 to define a “weak IV.”^31^ These parameters test the validity of the first IV assumption of MR, known as the relevance assumption, which states that the genetic variant is directly associated with the exposure.^32^ Weak IVs can bias the effect estimates derived from MR in the presence of confounding factors that may affect the exposure-outcome relationship. Results from this annotation are available in Table S3.

### Colocalization

For genes with a significant MR result, we performed colocalization between the outcome GWAS (or meta-analysis) and the *cis*-variants available from the QTL sources passing MR for that gene-trait using the coloc package^33^ in R. Marginal (unadjusted) eQTL/pQTL results and unconditional results on each of the instruments used in the MR were used. For GTEx instruments identified by conditional associations, since the full associations were available to us, the conditional results were used. We used variants with MAF >1% and a ±250-kb window around each of the instruments. We defined strong colocalization as having posterior probability for hypothesis 4 (PP.H4) > 0.8 (the probability of a shared causal variant) for at least one IV. We also noted when significant gene-trait pairs had strong colocalization for every IV. For case-control studies, we incorporated *p* values and the proportion of the samples that are cases in the outcome GWAS. For quantitative traits, sdY (standard deviation of the trait) was calculated using the variance of beta and MAF. Prior probabilities were set to default values.

### Assessment of druggability

We extracted drug information for protein targets from ChEMBL^20^ (version 34). For all protein targets, we acquired Ensembl IDs when available using UniProt’s REST application programming interface. For each drug where the information was available, we assigned all indications for those drugs and the clinical phase for that indication. Also added was the MoA for the drug and the interaction it has with the target. For this, we classified as positive modulation (activator) or negative modulation (inhibitor) or other. Drug information can be found in Table S4.

### Protein-protein interactions

Using the approach introduced by MacNamara et al.,^34^ we used protein-protein interactions (PPIs) to investigate associations between significant gene-traits and approved drug target-indications. We constructed a PPI network containing one-step interactions between all instrumented genes. The aggregated PPIs network was constructed containing seven different PPI resources: Complex,^35^ Lit BM,^36^^,^^37^ Metabase (https://portal.genego.com/), OmniPath,^38^ HIPPIE,^39^^,^^40^^,^^41^^,^^42^ HI union,^37^ and STRING.^43^ In this process, we used the 0.8 quantile of the STRING scores as the significance cutoff and only incorporated the interactions with scores within the top 20th percentile. A similar significance cutoff was calculated and applied for the HIPPIE dataset.

### Calculation of pairwise semantic distance between terms

Here, we describe the method and implementation for the calculation of pairwise semantic distance between trait terms. The dataset consists of trait terms from the following seven source groups: Clinvar; drug indications (ChEMBL34); GWAS Catalog; knockout (KO) models (Mouse Genome Informatics [MGI] database); genetic phenotypes from MVP, UKBB, and FinnGen; OMIM; and putative loss-of-function (pLOF) burden analysis from UKBB. We used ScispaCy^44^ (en_core_sci_lg version 0.5) as the encoder model for its ability to efficiently encode biomedical text and symbols, and for each of the terms we computed a 200 × 1 vector as the semantic vector representation of the term label. The generated encodings were then loaded into an Elasticsearch vector store as dense vectors, where the top-N candidates that are most closely associated with the input vector of a query term can be efficiently computed and retrieved on-the-fly using a k-nearest neighbor search based on a cosine similarity metric. For each of these trait terms, we then computed the cosine similarities between this trait term and each term of the seven source groups (including the group this trait term belongs to). We then retrieved for each trait term their 3% most similar trait terms in the semantic vector space for each source. Source code for the implementation is available under the “MRCIEU/phenotype-mapping” repository on GitHub (https://github.com/MRCIEU/phenotype-mapping/tree/2023-06-mvp-terms/analysis/pipelines/mvp_ontology_distance_round3).

### Novelty allocation using the GWAS Catalog

To assess one novelty aspect of our results we consulted the GWAS Catalog^45^ (downloaded with most recent update from January 30, 2023) to determine whether the gene-trait pair found to be significantly associated in our MR results had a gene/variant previously reported as associated with the phenotype in question. To map our phenotypes (phecodes) to the ontology-based phenotypes, we used a natural language processing (NLP) tool to help rank and assign matches between the description of the phenotype and Experimental Factor Ontology (EFO) terms. The full list of EFO terms used and their parent terms is found in Table S5. We also assigned parent terms, or high-level trait classification, by using the assigned EFO terms and ascending ancestor terms until we captured a predefined ontology term in our list (Table S6). Additionally, for each phenotype, we created a list of the top 3% closest phenotypes in the GWAS Catalog by the embedding method described previously.

For gene-trait pairs that passed two-sample MR, we selected the region corresponding to 250 kb before the TSS of the gene in question to 250 kb to after the Transcript End Site of the gene in question. We then searched the GWAS Catalog for either a direct match on ontology, a match on the parent term, or by a semantic distance match. For each type of match (self, parent, and distance) we provide a score—1 for a match on the region and 0 for no match and therefore novel. We wish to emphasize that this search scheme is not error proof. GWAS Catalog significant associations sometimes do not reflect the full body of literature linking genetic variants in a gene to a specific phenotype.

### Evaluating features associated with selected MR findings

To evaluate whether specific features of our data could be predictive of a selected MR gene-trait, we have created a file containing all selected gene-traits by MR (after the described filter steps) and the same number of non-selected MR results. All gene-traits (selected MR and non-selected MR gene-traits) were mapped to features thought to be predictive of a significant MR result (Table S7 provides a description of each engineered feature). Matching significant and non-significant results followed the same filtering steps as used for significant results, including checks on directionality and eQTL and pQTL sources. Specifically, for each of the final selected MR results, we randomly selected a result without robust evidence of causality with the same number of eQTL and/or pQTL sources to test for directionality of the estimated effect. The specific QTL source(s) selected to be represented in the non-selected MR result was a function of the number of sources represented in the positive result and a weighted probability reflecting the relative distribution of each QTL source in the overall tested MR gene-traits. The resultant file was then used to compare feature enrichment among significant MR results. Feature enrichment was always estimated using a logistic regression approach in which the outcome was being or not being a significant gene-trait in the MR analysis. Enrichment of each derived feature was tested using the above-described set of gene-traits in a logistic regression framework using as the dependent variable the status of the MR gene-trait (initially selected or non-selected) and as the independent variable each derived feature.

### Mapping orthogonal sources

To garner support for our significant gene-trait pairs, we sought to identify whether these connections had been previously uncovered in an assortment of sources of biological information. To determine whether our gene-trait pair had been reported at both the phenotype and gene levels, we observed OMIM,^46^ pLOF data from GeneBass,^47^ KO models from MGI,^48^ and variants of clinical significance from ClinVar.^46^^,^^49^ For each source, we assigned EFO codes for the phenotypes as well as the parent terms for the selected phenotypes. The OnToma package for Python was used to map OMIM and pLOF phenotypes to EFO codes and terms, MONDO codes and terms, and ORDO/Orphanet codes and terms. We have referred to any ontology terms from these systems as EFO codes. The OnToma package (https://github.com/opentargets/OnToma, version 1.1.0) supports two forms of input: phenotype descriptions such as “heart failure” and phenotype codes from non-EFO systems, such as OMIM IDs. The method then returns a list of EFO codes matching the input if any are found. OnToma mapping was also attempted with phenotypes from MGI, but only a small subset of phenotypes mapped successfully. Therefore, MGI phenotypes were mapped to EFO terms at the distance and parent levels only.

### Creating a database of approved drug targets and clinical indications for enrichment analysis of predictive features

The creation of a framework in which the enrichment of significant MR gene-traits, as well as other biological features, can be estimated and tested under a statistical framework was accomplished following several steps. Initially, we mapped all approved (phase 4) drug targets (genes) to unique clinical indications. This step was done manually, where all drug target and indication pairs from ChEMBL34 were clustered with similar pairs. At the end of this step, we were able to define 3,565 unique drug target-indications in ChEMBL 34. Second, we mapped all approved indications to the phenotypes with available genetic association summary statistics. These steps specifically avoided that rediscoveries or repurposing opportunities were counted multiple times (e.g., discovering the PCSK9 [MIM: 607786] association with dyslipidemia, with mean low-density lipoprotein [LDL] levels, and with the use of anti-lipidemic medications should count as a single rediscovery and not three distinct ones). Finally, we created a file containing positive and negative controls for training a model for approved drug indications. Positive controls are the approved drug targets-indications just described. Negative controls were paired to positive controls by the clinical indication (to avoid bias due to increased GWAS statistical power for commonly approved indications) and derived by randomly selecting a gene from the gene set with at least one significant genome-wide MR result (to avoid the bias of creating negative gene-trait pairs using genes with weak genetic instruments). For each existing positive control, we created 10 random negative controls. Our training dataset was composed of 3,565 positive controls and 35,614 negative controls.

### Model training and testing

In the conducted study, a supervised classification task was undertaken employing the XGBoost algorithm,^50^ facilitated through the xgboost package in R. The approach adopted involved defining a comprehensive parameter grid to facilitate the meticulous tuning of the model, aiming to optimize its predictive performance. The parameter grid encompassed a variety of hyperparameters, including the learning rate (0.01, 0.1, 0.3), maximum depth of a tree (3, 6, 9), minimum child weight (1), and the subsampling rate (1). Following the establishment of the parameter grid, the xgb.tree method was employed to implement the XGBoost model (objective function binary:logistic). A grid search cross-validation technique was utilized to systematically explore the hyperparameter space, ensuring that each unique combination of hyperparameter values was evaluated to ascertain the optimal model configuration. The model was designed to minimize the error (evaluating metric) in classification that a given gene-trait is an approved drug target and its indication. The performance of each model was evaluated using 5-fold cross-validation steps using 80% of our positive and negative controls from the benchmark drug indication file to train the model. A held-out sample of 20% of positive and negative controls was used in model testing and to establish the performance of the final model. This file was derived using 10 negative controls for each positive control. The model was tested and trained using the imbalanced dataset.

### Lipids vignette

To showcase the repurposing and rediscovery opportunities that our approach can identify, we selected lipids, a continuous trait that is well powered and has several known targets and approved drugs. Initially, we filtered the ChEMBL34 clinical indications to all terms pertaining to dyslipidemia treatment. For this we selected all indications matching the following EFO terms: “Abnormal circulating lipid concentration,” “Combined hyperlipidemia” (MIM: 144250), “Disorder of lipid metabolism,” “familial hypercholesterolemia” (MIM: 143890), “Hypercholesterolemia” (MIM: 143890), “hyperlipidemia” (MIM: 602491), “hyperlipoproteinemia” (MIM: 238600), “Hyperlipoproteinemia type 1” (MIM: 238600), “hyperlipoproteinemia type 3” (MIM: 617347), and “Hyperlipoproteinemia type 4” (MIM: 144600). Among these terms we observed 251 different drug indications with a clear molecular target mapped to an existing drug. For the recovery of lipids genes in our set of selected MR results we used the following phenotypes: Apolipo_A, Apolipo_B, CircCholMed, Disorders of lipoid metabolism, HDLC_Max, HDLC_Max_INT, HDLC_Mean, HDLC_Mean_INT, HDLC_Min, HDLC_Min_INT, Hypercholesterolemia, Hyperlipidemia, LDLC_Max, LDLC_Max_INT, LDLC_Mean, LDLC_Mean_INT, LDLC_Min, LDLC_Min_INT, Mixed hyperlipidemia (MIM: 144250), TotChol_Max, TotChol_Max_INT, TotChol_Mean, TotChol_Mean_INT, TotChol_Min, and TotChol_Min_INT.

Previous GWAS hits were obtained from Graham et al.^51^ (Tables S3, S5, and S18). Previous GWASs were derived from merging all unique genes annotated in Tables S3, S5, and S18 (2,507 genes) and then parsing this list to contain only protein-coding genes (1,236 genes). Protein-coding genes were obtained from www.ensembl.org on November 10, 2023. Genes with rare variants significantly associated with lipid traits were extracted from Selvaraj et al.^52^ (Table S4).

## Results

### General findings from a large-scale two-sample MR exploration on human disease

**Figure 1:**
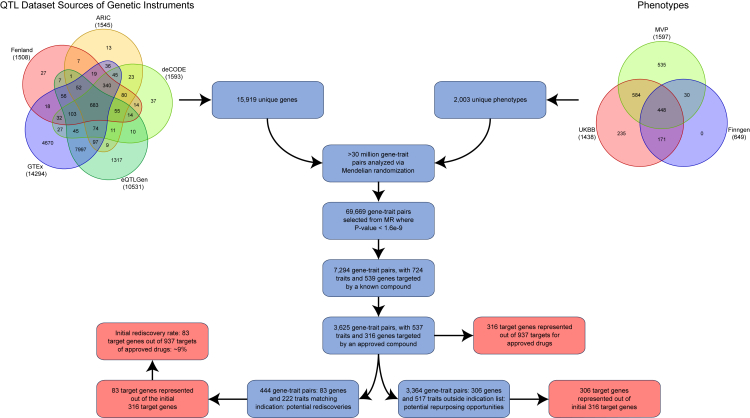
A flowchart demonstrating the main findings from the pipeline and the resulting counts of both rediscovery and repurposing opportunities

In this effort, we systematically studied 2,003 distinct phenotypes meta-analyzed from MVP, UKBB, and FinnGen encompassing summary statistics derived from more than 1.2 million individuals (Table S1). We analyzed each phenotype against 15,919 genes, instrumented through 15,700 transcripts (eQTLs derived from GTEx and eQTLGen) and 1,933 protein (pQTLs derived from ARIC, deCODE, and Fenland) levels through 2-sample MR. In total, we tested 31,525,236 unique gene/protein-trait associations (Figure 1). A detailed descriptive analysis of the genetic instruments used is available as Appendix S2.

From the 2-sample MR analysis results, we observed 108,107 gene-trait pairs with a *p* value ≤1.6e−9 (79,114 unique gene-traits). If the same transcript/protein was identified as potentially causal of the same trait using more than one instrument source, then we compared whether the predicted directionality of effect was concordant among the sources (i.e., among different eQTL, including different GTEx tissues, or among different pQTL sources). If there was discordance, then these gene-trait pairs were removed from consideration resulting in 69,669 unique gene (6,447)-trait (1,052) pairs (Figure S2; File S1) (henceforth referred to as selected MR results) selected for downstream analysis.

We systematically compared our results to those available in the GWAS Catalog^45^ (as of January 2023). Of the 69,669 selected results, 42,737 (∼61%) corresponded, in the GWAS Catalog, to a previously reported genome-wide significant association within the region (see material and methods) assigned to the same trait. We observed 26,932 (∼39%) causal effects that were not previously reported. That is, there was no significant result described in the GWAS Catalog for the same trait that mapped to the same genomic region. When considering traits mapped to a similar parental disease hierarchy, as opposed to the same trait, 8,932 (∼13%) of our selected results did not map to any association described in the GWAS Catalog for any trait in the same disease hierarchy. The described degree of results not previously reported when compared with available gene-trait association in the GWAS Catalog, together with the fact that MR explicitly instruments the relationship between a gene and a trait, highlight the advantages of our approach over a GWAS results-based approach.

### Triangulation of MR results with orthogonal sources to annotate findings

To further increase the confidence on our MR findings, we triangulate our MR hits with orthogonal sources such as OMIM,^46^ ClinVar,^49^ pLOF burden analysis of rare variants from the UKBB,^47^ and the MGI mouse knockdown database^48^ for gene-trait pairs that could be mapped to those tested in our study. To enable triangulation at scale of MR hits with orthogonal sources, systematic mapping of traits across all these sources is essential. Whereas mapping the genes is straightforward, mapping phenotypes among these databases and our study is more complex. To map phenotypes among these databases, we created for each trait, in each of the datasets, three different mapping schemes with the traits from the list of phenotypes we used in the MR analysis. These schemes were an exact match (same trait name or concept), a parent term match (where we mapped all the used traits to one or multiple parent terms pre-defined in a system level dictionary), and a semantic distance match (where terms were matched by a semantic distance approach using embeddings of all traits present in all used databases). We discuss in detail our phenotype mapping strategy in the material and methods section.

**Figure 2:**
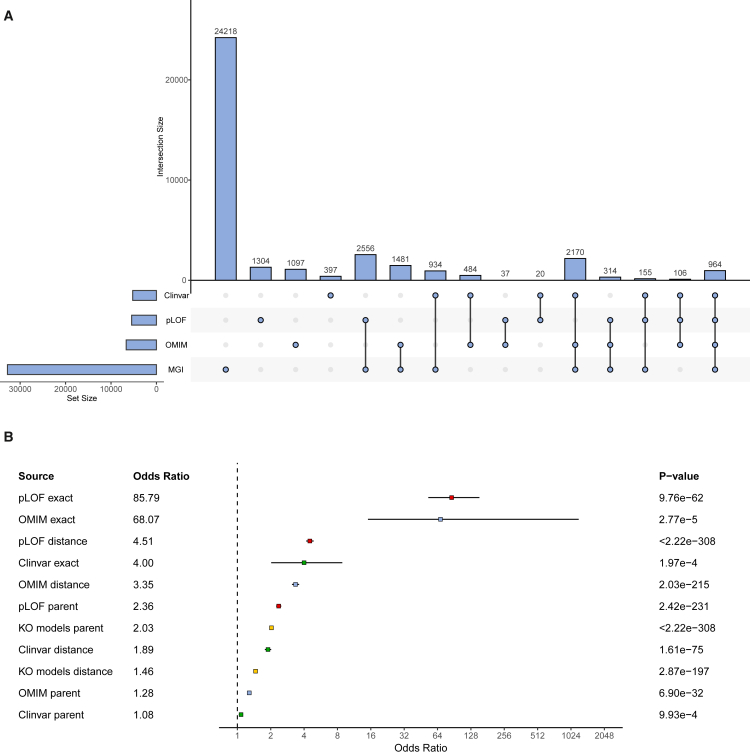
Plots demonstrating the overlap ands odds ratios of orthogonal sources (A) Upset plot representing the different intersection numbers of gene-traits among the used sources of orthogonal biological information. (B) Forest plot of the association between the different biological features as predictors of being a significant MR gene-trait. Different distance metrics used for capturing a trait match were exact, same EFO term for both MVP and biological database; distant, closest 3% terms in the ranked list between MVP and biological database using a semantic distance metric; parent, same parent term in both MVP and biological database. Error bars represent 95% confidence intervals.

We mapped all tested gene-trait pairs in MR to the curated catalogs of orthogonal biological information. Selected MR gene-traits were significantly more likely to be represented by all sources of biological information as compared with non-selected MR gene-trait pairs (Figure 2). This was observed for all schemes used for mapping phenotypes between datasets. For example, if the gene being tested for a specific trait has been previously described as a gene-trait association in OMIM, there is a 68-fold increased odds of the gene-trait being a selected MR result (*p* = 2.77e−5). Similar behaviors are observed if the gene-trait being tested has been identified in a pLOF rare variant burden analysis in the UKBB (odds ratio [OR] = 86, *p* = 9.76e−62) or described as a gene causing the trait in ClinVar (OR = 4.00, *p* = 1.97e−4). Interestingly, enrichment for KO models was lower than anticipated (OR = 2.02 and OR = 1.46 depending on the phenotype mapping strategy) (Figure 2; Table S8). Of note, the four described orthogonal sources were significantly independent; that is, they were all significantly associated with a selected MR gene-trait in a multivariable model adjusting for all orthogonal sources (Table S9).

Although these findings reinforce a putative causal role of the selected gene-traits, from a drug discovery translational perspective it would be ideal to quantify the odds of a selected gene-trait to be developed into a successful drug target. To answer this question, we used historical data from successful drugs with known efficacy targets.

### Mapping causal genes and traits to approved drugs and their indications

Using ChEMBL^53^ release 34 we mapped all selected gene-traits from our MR effort to existing drug targets and their indications. To this end, we have systematically mapped all drug targets described in ChEMBL 34 (1,533 mapped targets from 4,315 in clinical development or approved drugs) and disease indications (2,031 unique indications) into 33,102 unique drug target-indication pairs (File S2). From the 6,447 set of unique genes in the selected MR gene-trait results (the 69,669 gene-trait pairs described above), 539 were the target of an existing compound, including preclinical compounds (represented in 7,294 selected gene-traits) (Table S10). From these, 83 genes mapped to the target of an approved drug. This set corresponds to 9% of all described targets for approved drugs in ChEMBL 34 (a total of 937 approved drug targets described). Genes that are targets of drugs in development (listed as developmental phases 1–3) are significantly overrepresented in our set of selected MR gene-traits as compared with genes that are not targets of an existing drug (Table S10). Looking at potential targets that are currently listed specifically in phase 3 clinical development by ChEMBL 34, from the 7,294 selected gene-traits that target a drug in clinical development, 1,477 had their maximal phase listed as 3. From these, we were able to identify 183 selected gene-traits where the indication of the ongoing trial efforts matches the trait the gene was associated to our MR results (Figure S3; Table S11).

**Figure 3:**
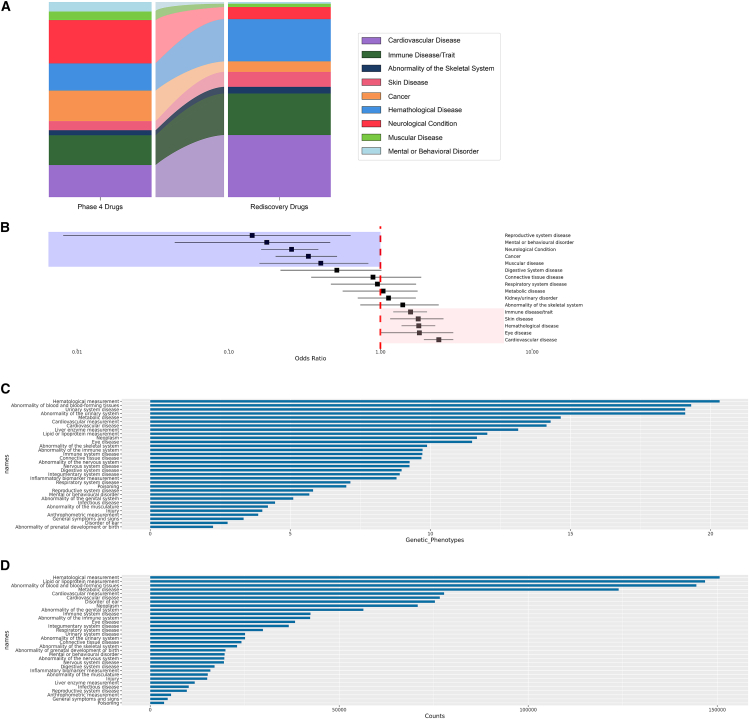
Results from genetic rediscoveries of approved drugs (A) Stacked bar plot of the distribution of parent terms for all currently approved drugs indications (left bar), and distribution of parent terms among gene-trait pairs considered rediscoveries (right bar). (B) Forest plot representing the over- or underrepresentation of specific parent terms among rediscoveries. The error bars represent 95% confidence intervals. (C) Mean number of available genetic phenotypes per parent term indication. (D) Mean number of cases used to calculate the association between genetic variants and the outcome per parent term.

From the 3,625 gene-traits with genes that were targets of approved drugs, we mapped 257 selected gene-traits (70 unique indications) that fully recapitulated both the approved drug target and its approved indication. We consider these gene-trait rediscoveries, and they represent 83 unique drug targets covering 9% of all approved drugs targets (2.74% of all approved target indications were rediscovered) (Figure 3). Using the MR estimate and the approved drug MoA, we compared within the set of rediscoveries whether the calculated MR estimate direction of effect was in agreement with what would be predicted by considering the approved drug MoA. The MR estimated direction of effect correctly predicted the expected MoA 84% of the time (*p* = 4.46e−9, 95% confidence interval [CI] 74%–92%). We did not observe an association between being identified through a pQTL and a higher odds of correctly predicting the expected MoA (*p* = 0.47, odds of correctly predicting MoA for eQTLs 84%, odds of correctly predicting MoA for pQTLs 89%).

Rediscoveries were notable for a range of different diseases and organ systems (Table S12). Nonetheless, by comparing the drug indications using a systems-based approach, we observed that drugs targeting some biological systems were overrepresented as compared with the distribution of all approved drugs and their indications. Specifically, drugs targeting cardiovascular system diseases (OR = 3.3, *p* = 5.31e−4) were more likely to be rediscovered through our approach, whereas drugs targeting different types of cancer (OR = 0.2, *p* = 2.88e−13) or neurological conditions (OR = 0.1, *p* = 1.22e−5), among others, were less likely to be rediscovered (Figures 3B and 3C). We explored technical variables that could explain a higher or lower rediscovery rate of drugs targeting specific organ systems such as the number of mapped phenotypes between GWAS data and approved drug indications, the total number of approved indications for a particular organ system, and predictors associated with the statistical power of available GWAS (such as the total number of cases). The most important predictor was the percentage of mapped phenotypes with <10,000 cases (*p* = 0.003), suggesting that the underrepresentation of rediscoveries for some disease types might be mitigated by increasing the number of GWASs (and, consequently, the number of cases) used in the MR analysis for some specific diseases such as cancers and neurological and psychiatric conditions in future iterations of this work (Table S13).

### Potential repurposing opportunities observed for approved drug targets

**Figure 4:**
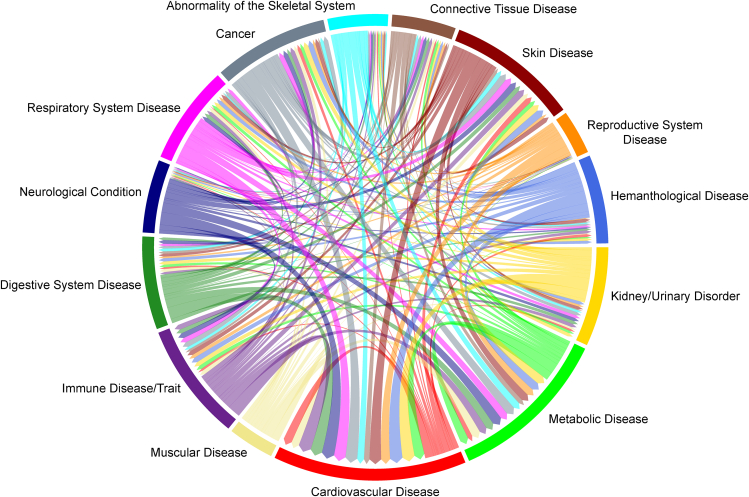
Circle plot representing the flow of disease categories for approved drug indications to the disease categories of potential repurposing opportunities based upon our findings

A corollary of the mapping of selected MR gene-traits to approved drug targets is the possibility of unraveling drug repurposing opportunities. These are the selected gene-trait pairs mapped to a gene that is the target of an approved drug but not associated with a trait in the listed approved indications for that drug. Rather, the associated trait is a novel one putatively modulated by the approved drug. Of the 3,625 selected gene-traits that mapped to an approved drug target, 3,364 had a trait different from the one described as an approved indication in ChEMBL34. These encompass 1,589 different drugs and 960 unique potential repurposing (354 indications) or safety (236 indications) concerns for currently approved drugs. In Figure 4, we show the transition between approved indication disease systems and repurposing disease systems. A complete list of all observed potential repurposing opportunities can be found in Table S14.

Among the several observed repurposing opportunities, some might deserve special consideration due to the prevalence of the indication and/or the wide usage of the approved drug: probucol, an ABCA1 (MIM: 600046) inhibitor with lipid-lowering properties for glaucoma; galcanezumab, a CALCB (MIM: 114160) inhibitor approved for migraine prophylaxis for obesity (MIM: 618406); anrukinzumab, an interleukin-13 (IL-13) (MIM: 147683) antagonist approved for ulcerative colitis (MIM: 266600) repurposed for psoriasis (MIM: 177900); tocilizumab, an IL-6R (MIM: 147880) antagonist approved for rheumatoid arthritis (MIM: 180300) repurposed for atrial fibrillation (MIM: 608583); and metformin, an NADH dehydrogenase inhibitor approved for type 2 diabetes (MIM: 125853) repurposed for atrial fibrillation (MIM: 608583).

It is important to note, however, that these initial repurposing opportunities should be contemplated considering the potential misassignment of directionality given by blood-based eQTLs and pQTLs^54^; a comprehensive examination of safety concerns is beyond the scope of the present work. One example that can be highlighted from our results is the presence of trastuzumab, an ERBB2 (MIM: 164870) inhibitor, as a potential repurposing opportunity for both atrial fibrillation and heart failure (Table S14). Although there is the possibility that trastuzumab might be protective against both conditions in specific scenarios, clinical knowledge highly suggests the contrary. Trastuzumab is a known cardiotoxic compound,^55^ and most probably the highlighted signal should be classified as a safety concern rather than as a repurposing opportunity.

### Selected MR gene-trait pairs are predictors of approved drug indications

A mapping framework that triangulates data from four key areas—gene targets of existing pharmaceuticals, their approved therapeutic uses, results from GWASs, and available genetic proxies of molecular exposures—can be employed. This framework serves as a foundation for developing and validating predictive models for MR analyses of gene-trait associations using the gene targets and indications of currently approved drugs. With this in mind, we have mapped approved drug targets to unique clinical indications (to reduce bias due to similar clinical indications for the same drug target), mapped approved indications to the phenotypes with available genetic association summary statistics, and created a benchmark file containing positive and negative controls for training a model for approved drug indications. Negative controls were paired to positive controls by the clinical indication (to avoid bias due to increased GWAS statistical power for commonly approved indications) and derived by randomly selecting a gene from the gene set with at least one significant genome-wide MR result, to avoid the bias of creating negative gene-trait pairs using genes with weak genetic instruments. For each existing positive control we created 10 random negative controls. Our training dataset was composed of 4,027 positive controls and 40,216 negative controls. Our results show that an MR result with robust evidence of a causal effect is associated with a 2.77 (95% CI 2.3–3.4) higher odds of the associated gene-trait being a positive control (i.e., one of an approved drug indication) (*p* ≤2e−16). This estimate is concordant with previously published estimates using a smaller number of phenotypes.^10^^,^^56^^,^^57^^,^^58^^,^^59^ Acknowledging that other criteria can be used to select gene-trait pairs for further analysis and that depending on the used criteria the sensitivity and specificity of the approach can significantly change, we provide in Appendix S3 a comparison of different selection criteria and the resultant number of selected gene-trait pairs, rediscoveries, and relative odds of a selected gene-trait to be an approved drug target and indication.

### Genetic colocalization between GWAS and instrument signals improves specificity and the positive predictive value at the cost of reducing sensitivity for detecting approved drug targets and their indications

Prior work on the use of MR for early target identification has used colocalization as a filtering step after the initial MR result.^60^^,^^61^ To assess the use of genetic colocalization within this strategy, we evaluated the impact of using colocalization for filtering selected MR results (that is, accepting as potential new targets those that have an MR *p* <1.6e−9 and also significantly show a high colocalization signal, defined as selected MR results with at least one PPH4 region (both traits share a single causal variant within a genomic window defined around an instrumented variant) colocalization signal >0.8 per instrumented gene/protein.

As expected, using genetic colocalization as a filter reduced the number of selected MR results from the initial 69,669 gene-trait pairs to 54,636 (78%) gene-trait pairs when at least 1 significant PPH4 result was required.

The consequence of applying this filter was a reduced number of rediscoveries but also an increase in the enrichment associated with a selected gene-trait. While gene-traits only passing MR were associated with an OR = 2.77 (95% CI 2.3–3.4) of being an approved drug indication, restricting selected MR results to only those passing at least 1 colocalization test defined a set of gene-traits associated with a 3.09-fold enrichment (95% CI 2.5–3.8, *p* = <2e−16). The price paid by this increased positive predictive value was a reduced sensitivity: from an initial set of 148 selected gene-traits rediscoveries unique indications, 109 were observed when requiring at least 1 colocalization result.

With these results we posit that the reduced sensitivity could significantly impact the potential for new discoveries. Therefore, we decided not to use genetic colocalization as a required criteria for downstream analysis of selected MR results, but rather as a potential predictor of a selected gene-trait to be a true positive (see subsection Model training and testing).

### Orthogonal information is predictive of approved drug targets and indications

Using the same strategy to show the capacity of a selected MR result to predict an approved drug target-indication, we inquired whether the trait mapping schemes and secondary sources of biological information we previously described as associated with a selected MR gene-traits could also inform on the odds of a particular gene-trait being the target of an approved drug indication. Gene-traits that could be mapped to any of the different biological databases (and in any of the three trait mapping schemes used) were significantly more likely to be approved drug targets and indications (Table S15).

### Understanding MR-identified target landscape with protein interaction networks

There has been interest in the potential to extend the space of credible drug targets by examining proteins that interact with “genetically validated” targets. Such targets may be preferred to the original because they have improved properties (e.g., with respect to safety or chemical tractability).^62^ Here, we tested whether information from the protein interactome of a selected MR gene-trait pair could inform the predictive value of it being associated with an approved drug for the same gene-trait pair. This was done by an expansion and modification of a similar approach used to define the enrichment of MR results within the set of approved drug indications.^29^ Specifically, we constructed aggregated PPI networks containing seven different PPI resources: Complex,^35^ Lit BM,^36^^,^^37^ Metabase (https://portal.genego.com/), OmniPath,^38^ HIPPIE,^39^^,^^40^^,^^41^^,^^42^ HI union,^37^ and STRING^43^ (details on pre-processing and assembly can be found in material and methods). Using the aggregated network, we observed that the number of first-degree protein partners was higher in selected MR gene-traits as compared with gene-traits that did not reach statistical significance (*p* < 2e−16). More important, the PPI information was predictive of associations between a gene-trait pair and an approved drug for the trait. Namely, having a significant MR result and being a gene that is a PPI with another gene that also has a significant MR result for the same trait increase the odds of the queried gene-trait being an approved drug target and indication (OR = 1.89, 95% CI 1.8–2.0, *p* ≤ 2e−16). In addition, the odds of a given gene-trait pair being associated with an approved drug was significantly higher (OR = 1.17, 95% CI 1.1–1.3, *p* = 1.5e−5) when a first-degree PPI of the gene was associated with an approved drug for the same trait compared with when no PPIs of the gene were approved drug targets for the trait. Interestingly, the different PPI resources did not share a large amount of specific PPIs (see Figure S4) and, hence, their value to the overall enrichment was mostly additive (see Table S16 for a fully adjusted model). As expected, having a first-degree PPI of the gene associated with an approved drug for the same trait was positively associated with higher odds of the gene-trait being mapped to an approved drug indication in most used PPI resources., Unexpectedly, HI union, a PPI resource derived mostly from cancer cell lines, was significantly and independently associated with a reduced odds of the initial gene-trait being mapped to an approved drug indication.

A second strategy that we devised aiming at using information provided by PPI was to determine whether a first-degree partner of a selected MR gene-trait was also a selected MR gene-trait to the same trait and whether this information influenced the odds of the initial gene-trait to be mapped to an approved drug indication. For this analysis, we recursively tested more than 19 million gene-trait combinations and calculated the number of times a first-degree protein partner had robust causal evidence to the same trait of a given gene-trait pair. Results showed that selected MR gene-trait pairs were enriched for the presence of a first-degree protein partner that also had significant MR results for the same trait, independent of the total number of interacting proteins of the gene. Having a first-degree protein partner with a significant MR result for the same trait significantly increased the odds of being a selected MR result by 22-fold (*p* < 2e−16). In addition, similar to the discussion above regarding the case of protein partners being a target of an approved drug, for a given gene-trait pair, having protein partners associated with the same trait also significantly increased the odds of the initial pair being the target and indication of an approved drug. The larger the number of protein partners associated with the trait of a given gene-trait pair, the higher the odds of the gene-trait pair being the target of an approved drug indication (e.g., each added protein partner associated with the same trait increases by 4% the odds of the initial gene-trait of being an approved drug indication [*p* < 2e−16]). This reinforces the intuition that convergence of multiple genetic associations on a pathway identifies key disease-causing pathways.

### Ranking selected MR results by the probability of success in drug development

**Figure 5:**
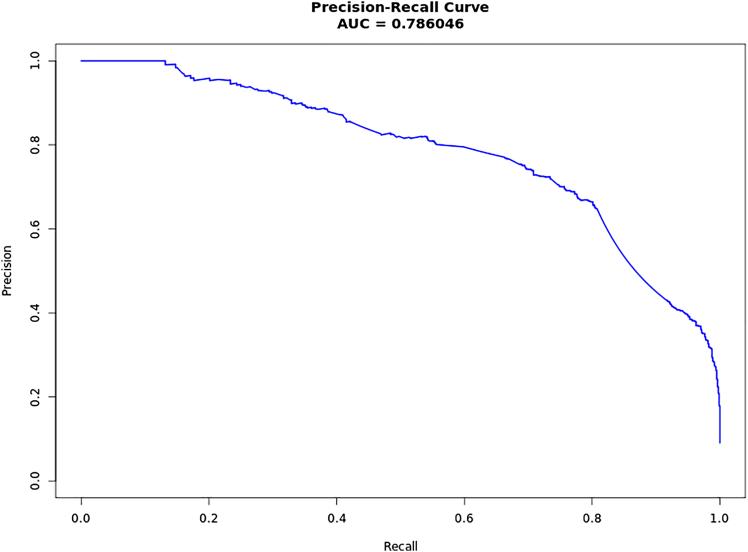
Precision-recall estimate of our classifier

From the 69,669 unique gene-trait pairs identified by MR, only a very small number were considered rediscoveries (i.e., a selected gene-trait pair that can confidently be labeled as a true positive finding). The great majority of selected gene-trait pairs described here, although potentially interesting, are still suggestive at best of a viable drug target and clinical indication. Leveraging the previously described resources and phenotype mapping strategies, we used the approved drug indications in ChEMBL 34 to derive a classifier able to predict the likelihood of a given gene-trait to be developed into an approved drug indication. For this, we used an ML model approach and engineered 40 features based on the different enrichments we described between a selected MR result and approved drug targets and indications (details of each engineered feature can be found in Table S7). We describe the modeling approach in detail in the Model trainining and testing subsection of Material and methods. Briefly, our classifier was trained using 80% of the entries in the previously described benchmark dataset and tested in the remaining 20%, which was kept as a holdout set only used to test the final model. We selected the most influential features and integrated these into a classifier for approved drug indications. The overall accuracy (Figure 5) was observed in a precision-recall area under the receiver operating characteristic curve (AUC) of 0.79. Additional characteristics of the model performances can be found in Appendix S4 and Table S7.

Using this model, we derived predicted probabilities for all 69,669 selected gene-trait pairs (File S1). Finally, we ranked all selected gene-trait pairs based on the probability described by this model. This type of data representation is now able to be used to compare selected MR gene-trait pairs regarding their similarity to gene-trait pairs of approved drug targets and indications.

The predicted probabilities derived from our model provide the possibility of being more restrictive when selecting MR results for downstream exploration. For example, using a very restrictive cutoff probability in which both the positive and negative predictive values are above 0.8, we still have 338 gene-traits (Table S13).

### Prioritization of targets for drug development identifies ANXA2 as a target for dyslipidemia

Among the many uses of the developed framework and models, one is their utilization in prioritizing new drug development programs.^63^ In fact, the probabilistic nature and output of our classifier permits that among different preexisting gene-trait candidates, one can identify the most promising lead to an approved drug indication. Here, we exemplify this approach describing our results for anti-lipidemic and anti-obesity targets.

Using ChEMBL 34, we identified 9 approved targets for treating dyslipidemia in humans. From these, our MR approach was able to rediscover three targets: *HMGCR* (MIM: 142910), *PCSK9* (MIM: 607786), and *NPC1L1* (MIM: 608010). We also identified 11 targets among the 36 targets that were or are being tested for dyslipidemia (i.e., are below phase 4, but are in clinical testing phases 1–3). Additionally, using 25 different lipid traits that are part of our effort, we observed 4,472 selected MR results (892 unique genes associated with at least 1 lipid-related trait). Among 1,236 genes previously annotated to be associated with a lipids GWAS hit,^64^ we identified 291 (33%) in our list of selected MR results that were also associated with lipids. From the protein-coding genes with rare variants previously associated with lipid traits (121), we were able to recover 81 (67%) of the total described. Considering all previously associated genes from both GWAS and rare variant analysis, and assuming a genomic window of 100 kb around each gene, we defined 750 unique genomic loci with at least 1 previously described lipid gene. Our results were able to map at least 1 MR-associated gene to 291 of these loci (39%). From the 291 we were able to map at least 1 of the previously mapped genes in 199 instances. Interestingly, in 136 of previously mapped lipids loci, our results suggest at least one other gene as the causal effector of inter-individual differences in lipid levels in these genomic intervals (Table S17). In addition, for loci where several genes were mapped as potentially causal for regulating lipid levels, the use of our ranking model was able to suggest the best candidate among associated genes. For example, on chr14:91865991-92140896, previous GWAS observation pointed toward *TRIP11* (MIM: 604505) as a lipids-related gene.^65^^,^^66^ We did not find causal evidence for *TRIP11*. Nonetheless, *ATXN3* (MIM: 607047) was an MR hit with robust causal evidence in the same genomic interval. Our ranking model classified *ATXN3* as a gene with a high probability of being a drug target for lipids. From the selected MR results associated with lipids that were neither previously described nor mapped to one of the previously described loci, we have identified 369 novel genes. Tuning our ranking model to a positive predictive value of 0.70, we identified 7 novel genes with high probability of being targets for drugs modulating lipid levels: *ANXA2* (MIM: 151740), *TNF* (MIM: 191160), *HAVCR2* (MIM: 606652), *MPIG6B* (MIM: 606520), *HIST1H1C* (MIM: 142710), *TMEM57* (MIM: 610301), and *GSDMC* (MIM: 608384). Of particular interest is the presence of annexin A2 (ANXA2) in the list, a natural inhibitor of PCSK9 (MIM: 607786) and an endogenous regulator of LDL receptor degradation.^67^^,^^68^

The evidence supports the results of our MR analysis and predictive model and suggests that our data may disclose several novel therapeutic targets for a wide range of human disorders.

## Discussion

Here, we have systematically leveraged genetic association results from a large number of phenotypes and integrated different database sources to generate almost 70,000 potential causal effects of genes/proteins on disease-relevant traits. This endeavor extends previous efforts of using genetic information for early therapeutic target identification by almost 10-fold^24^^,^^69^^,^^70^^,^^71^ and provides a new resource for investigation of the polygenic causes of a wide range of human phenotypes. By increasing the breadth of our gene/phenotype matrix of causal effects, we allow for a more diverse exploration of human disease diversity, as well as the therapeutic opportunities that might be revealed by using empirical population genetic evidence.

Initially, it is imperative to highlight the importance and challenges of highly diverse large-scale biobanks and data source integration.^72^ This was one of the main challenges of the current work. In fact, there are significant challenges associated with the mapping and integration of large-scale biobanks developed in different medical systems (MVP, UKBB, and FinnGen), genetic instrument resources derived from different research programs (GTEx, eQTLGen, ARIC, Fenland, and deCODE studies), different bioinformatic databases (OMIM, MGI, ClinVar), and different sources of experimental molecular findings (PPI networks from different studies). For this, we have approached each biological data source as a different construct and proposed different mapping schemes at the molecular and phenotype levels. At the molecular level we have opted for a gene-based approach, which is facilitated by using an MR framework; at the phenotype level, mappings can be more nuanced and difficult. Here, we opted for a hybrid phenotype mapping approach that used both the specificity of ontology-based classifications (e.g., EFO terms) and the more fluid capacity that medical term embeddings allow for. On top of this, we have been able to manually curate all different disease/trait-associated databases and map each of their terms to pre-defined parental terms (see Table S6) that allowed for more general disease classifications. The challenges posed by phenotype harmonization and annotations were paramount, given the disparate data products and annotation schemes in biology, not to mention the variations in model organisms and their associated phenotypes. Despite the availability of ontologies such as Systematized Nomenclature of Medicine (SNOMED), Fast Healthcare Interoperability Resources (FHIR), and the ICD coding system, which are increasingly utilized in medicine, they fall short in addressing the complex needs of our study—mapping different sources to a common denominator and understanding the phenotypic distances within and across sources. Our use of a text-embedding vector store, constructed through the text vector representation of disease names using a semantic similarity algorithm, emerged as a pragmatic solution. This approach successfully bridged the gap between the disease name/concept and higher structured disease hierarchies such as organ systems. Furthermore, the association estimates between the different distance metrics aligned with our expectations, validating the efficacy of our methodology. Although several prior tentative methods of integrating genetic, animal models, and molecular interaction data, coupled with the sophisticated mapping of phenotype hierarchies, have been used in specific biological and medical contexts,^10^^,^^59^^,^^73^^,^^74^^,^^75^ here, we provide an approach that is anchored by large-scale population genetic evidence.

The links created in our work are mostly based on a gene/protein level causal effects defined by MR. This approach relies on the duality defined by, on the one hand, the reliance on accurate genetic association statistics for the studied phenotypes and on the other hand, the capacity to capture unbiased interindividual variability on genetically determined exposures. Our approach has original contributions on both sides. On the outcome side, we are querying genetic association data from >2,000 different human phenotypes. Whenever possible, sample size and statistical power were maximized by meta-analyzing the largest genetic biobank studies currently available: the UKBB, the FinnGen study, and the MVP. This effort is reflected in the large diversity of available phenotypes for analysis and on the mean number of cases per phenotype analyzed. On the exposure side, we have used five different sources of genetic instruments, capturing both transcript and protein levels for many molecular entities. In addition, our instrument selection process only selected conditionally independent *cis*-instruments, thus reducing the chances of bias due to both model misspecification and horizontal pleiotropy. Nonetheless, it is worth noting that horizontal pleiotropy is difficult to avoid for different genes/proteins (particularly when overlapping or in proximity), which can be regulated by a common subset of genetic instruments or confounded by linkage disequilibrium. This makes the dissection at this level particularly challenging, especially for an effort conducted at the present scale. Despite this limitation, the tunneling of our inferences at the gene level, as opposed to at the variant level, which is usually the case for GWAS or PheWAS efforts, has the advantage of reducing the dimensionality of the results dataset and to add an extra layer of support for target identification.

Instead of focusing our inference to only transcripts or proteins, we decided to use several independent genetic instrument sources and contemplated the challenge of making inferences for the entire set of protein-coding genes in the human genome. Notably, there was very high correlation between the estimate of effects within the three pQTL sources and within the two eQTL sources, despite the use of different genetic instruments among the different sources (Figure S1). The correlation of predicted effects between eQTLs and pQTLs were, expectedly, smaller. Despite being comprehensive, this approach has clear limitations due to all the situations in which transcripts and proteins operate in distinct directionalities. As a result, it is still hard to define a particular effect that should be emulated in a new drug. A cautionary tale is that although the predicted directionality of effect was correctly predicted in approximately 85% of the instances, in about 15% we have significantly identified the correct gene-trait pair, but our results pointed in the contrary direction. A direct comparison between the different sources of genetic instruments was beyond the objectives of the present work and might prove challenging because of the very different nature of the study designs, technologies, statistical power, and biological relevance of each source. We believe this is a fruitful new avenue^76^ of research that should be explored in future analyses and provide the results that would be observed by our approach if we used only eQTL or pQTL sources (Appendix S3). It is also important to note that powerful new sets of genetic instruments for molecular phenotypes are being developed such as the novel UKBB proteomics dataset.

Drug rediscovery, in this paper, means identifying a gene-trait association that corresponds to one of the known efficacy targets of an approved drug (as the gene) and is linked to the same medical indication or therapeutic use for which the drug was originally approved (as the trait). Essentially, it is discovering genetic evidence that supports the target and indication of a drug. We were able to rediscover approximately 9% of known approved drug targets in ChEMBL 34 (Table S12). Some drug indications were rediscovered several times—that is, in several different tested phenotypes—and about half of all rediscoveries have not been described as a gene-trait associated in a previous GWAS study—that is, where the drug indication was previously supported by genetic evidence from common variants’ genetic association. As such, the concept of rediscovery reaches beyond the replication of previous genetic associations of genes and phenotypes that are drug targets and trait indications. These facts highlight both the power of the approach and the potential to still be leveraged in its future iterations. Of particular importance, because we have benchmarked the different steps of our procedure using a list of curated approved drug indications, we can use this information to guide new methods, data extraction, and needs of the current pipeline so that new iterations can be more sensitive and specific. This might be exemplified in the set of technical predictors of rediscoveries. We showed that for some specific disease categories, the number of explored phenotypes and case counts is still a bottleneck that needs to be surpassed. Another feature of note is the capacity to use continuous variables as proxies for disease/drug indications. For example, most of the enrichment in the rediscovery rate of cardiovascular drugs can be traced to anti-lipidemic phenotypes that can be captured by lipid and blood pressure measurements, two continuous variables available in all three used biobanks. Finally, further investigation is needed to determine whether the same genetic information used to establish significant MR effects can also be employed to stratify individuals based on the varying effectiveness of drugs identified through rediscovery signals.

Building on the insights gleaned from the analysis of 69,669 unique gene-trait pairs identified, our approach has effectively distinguished a small subset of rediscoveries, affirming their status as true positive findings. Nonetheless, most significant gene-trait pairs, although intriguing and potentially indicative of viable drug targets and clinical indications, are still in a preliminary stage. Leveraging the comprehensive resources and phenotype mapping strategies previously outlined, we have crafted a classifier, utilizing a list of approved drug indications from ChEMBL 34, to predict the likelihood of a given gene-trait pair progressing to an approved drug indication. Through the application of XGBoost, the classifier exhibited a precision-recall AUC of 0.79. At this point, it is important to highlight the conceptual limitation of our model, which is maximizing the selection of gene-traits that have a feature profile similar to that of approved drugs and their indications. Factors modulating a successful drug program are determined by many more factors than only the causal association between a gene product and a disease, and this should be taken into consideration when interpreting our results. In addition, some of the features used in our classifier might also be biased by the prior existence of approved drugs within their molecular pathways, such as the use of PPI information.^59^ However, the derived predicted probabilities for the extensive list of gene-trait pairs with robust evidence of a causal role now provide a nuanced and highly granular means of comparison and constitute a helpful resource to identify the most promising candidates for downstream exploration.

## Data and code availability

Full summary statistics of all the two-sample MR are publicly available and can be downloaded at the Department of Veterans Affairs’s Centralized Interactive Phenomics Resource (CIPHER) web portal (https://phenomics.va.ornl.gov/) or at https://osf.io/g8hjz/overview. All supporting annotation files are also available by request at CIPHER. The code used in this paper can be located at the following GitHub repository: https://github.com/BrianFerolito/MVP_efficacy.

## Acknowledgments

This research is based on data from the Million Veteran Program, Office of Research and Development, Veterans Health Administration, and was supported by award no. MVP000. This publication does not represent the views of the Department of Veterans Affairs or the US government. The full MVP acknowledgment can be found in File S3. This research used resources from the Knowledge Discovery Infrastructure at the Oak Ridge National Laboratory, supported by the Office of Science of the US Department of Energy under contract no. DE-AC05-00OR22725 and the Department of Veterans Affairs Office of Information Technology Inter-Agency Agreement with the Department of Energy under IAA no. VA118-16-M-1062. We would like to acknowledge the time and effort of the study participants and researchers in the Fenland study (https://doi.org/10.22025/2017.10.101.00001; https://www.mrc-epid.cam.ac.uk/research/studies/fenland/). J.C.W. is funded by the UK Medical Research Council via programme grant no. MC_UU_00002/18.

## Author contributions

B.R.F., J.P.C., and A.C.P. conceived and designed the project. I.A.S., B.Z., A.R.L., M.G., Y.T., F.H., H.I., and M.A.K. consulted and helped to develop drug-related data. C.G., D.R., and H.D. curated and assembled the QTL data. B.R.F., D.J.G., A.C.P., G.M.P., H.D., Y.L., G.P., G.G.T., G.B., and K.G.-P. developed code for various modules of the pipeline. J.C.W., A.R.V.R.H., K.C., J.M.G., T.G., and G.H. provided feedback on the experimental design. A.S.B., C.L., J.C.W., E.D., and L.E.S. provided substantive feedback on the manuscript. S.M., J.M., J.E.H., S.D., S.W., K.P.L., T.C., and P.S.T. provided administrative and material support. R.M., L.C., and N.K. helped plan and execute the project. All authors read and approved the manuscript.

## Declaration of interests

A.S.B. reports grants outside of this work from AstraZeneca, Bayer, Biogen, BioMarin, and Sanofi. M.G. is a full-time employee of Regeneron Genetics Center; her main contributions occurred while she was an employee of Open Targets. J.P.C. is a full-time employee at Novartis Institutes for Biomedical Research; his main contributions to the project occurred while employed at the VA Boston Healthcare System. M.A.K. is a full-time employee at Variant Bio; his main contributions occurred while he was an employee of Open Targets.

## Web resources

CIPHER, https://phenomics.va.ornl.gov/mvp-drug-discovery/
Online Mendelian Inheritance in Man, https://www.omim.org/

## Supplemental information

Document S1. Figures S1–S10, Appendices S1–S5, and File S3

Data S1. Tables S1–S17

File S1. Significant Gene-Trait Pairs

File S2. Processed Chembl34 Data

Document S2. Article plus supplemental information
