# Supplementary material for: Leveraging large-scale biobanks for therapeutic target discovery
Source: HGG Adv. 2025 Dec 9;7(1):100556. doi: 10.1016/j.xhgg.2025.100556 (PMC12799792; doi:10.1016/j.xhgg.2025.100556)
Supplement: Document S1. Figures S1–S10, Appendices S1–S5, and File S3 [file mmc1.pdf]

HGGA, Volume 7

Supplemental information

Leveraging large-scale biobanks

for therapeutic target discovery

Brian R. Ferolito, Hesam Dashti, Claudia Giambartolomei, Gina M. Peloso, Daniel J. Golden, Kai Gravel-Pucillo, Danielle Rasooly, Andrea R.V.R. Horimoto, Rachael Matty, Liam Gaziano, Yi Liu, Ines A. Smit, Barbara Zdrazil, Yakov Tsepilov, Lauren Costa, Nicole Kosik, Jennifer E. Huffman, Gian Gaetano Tartaglia, Giorgio Bini, Gabriele Proietti, Harris Ioannidis, Mohd A. Karim, Fiona Hunter, Gibran Hemani, Adam S. Butterworth, Emanuele Di Angelantonio, Claudia Langenberg, Maya Ghoussaini, Andrew R. Leach, Katherine P. Liao, Scott Damrauer, Luis E. Selva, Stacey Whitbourne, Philip S. Tsao, Jennifer Moser, Tom Gaunt, Tianxi Cai, John C. Whittaker, Million Veteran Program, Juan P. Casas, Sumitra Muralidhar, J. Michael Gaziano, Kelly Cho, and Alexandre C. Pereira

Supplementary Figures

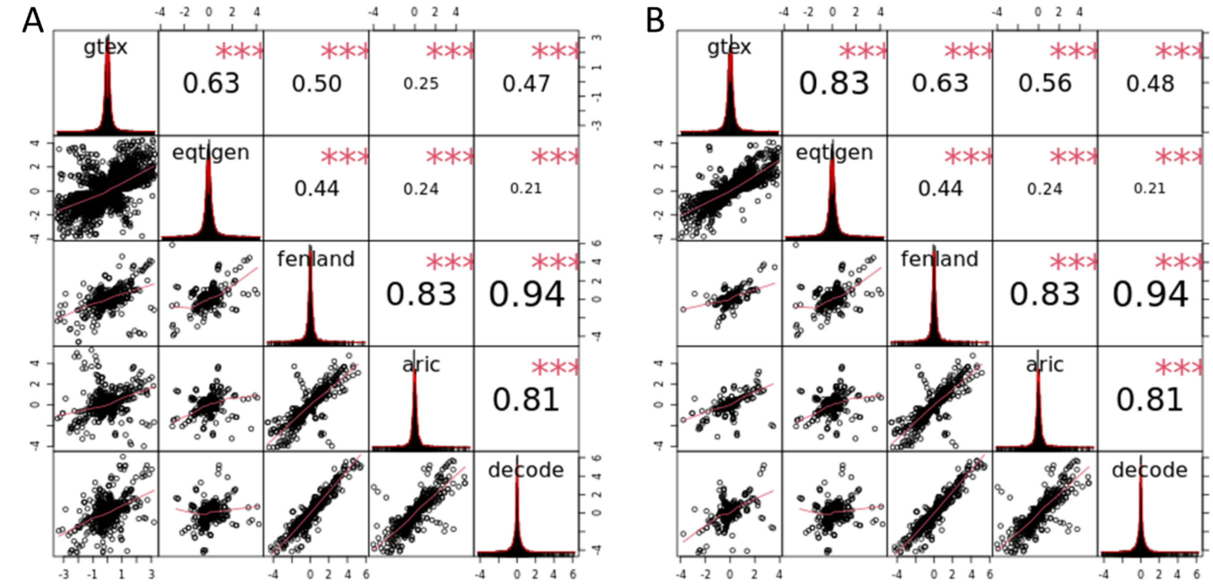

Figure S1: CorrFigure 1: Correlation matrices among the estimated MR coefficients for statistically significant traits, with panel A including all data from GTEx tissues and panel B including data only from GTEx whole blood.

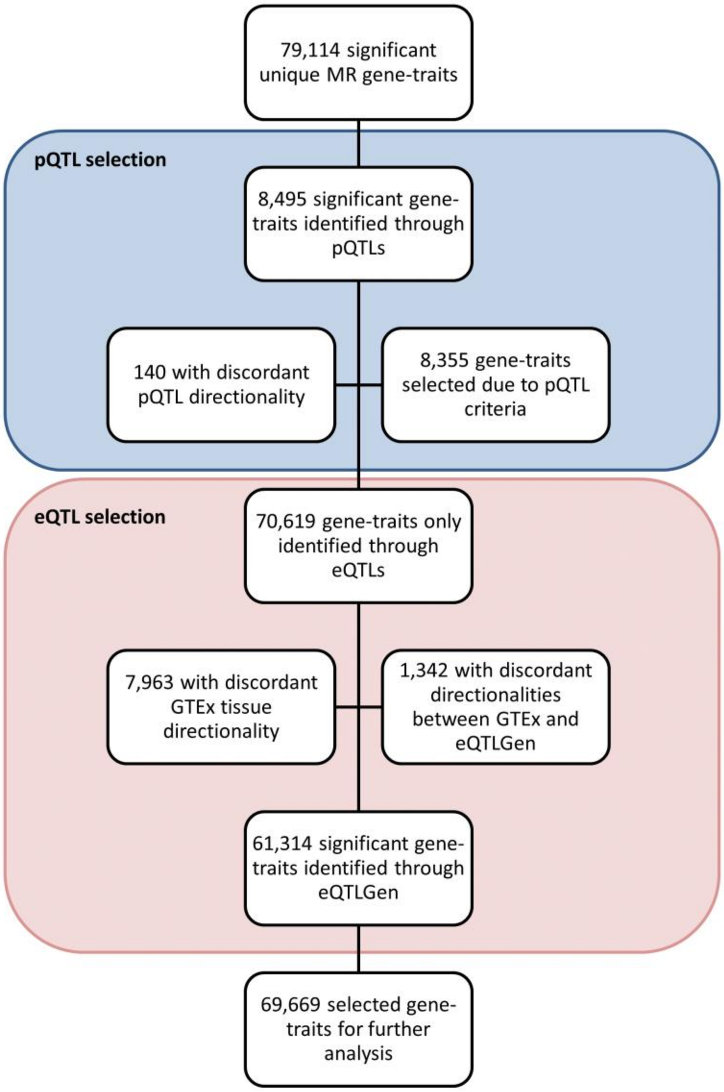

Figure S2: Flow chart showing numbers of significant MR gene-trait pairs identified through pQTLs versus eQTLs, with additional detail pertaining to discordant and concordant directionalities.

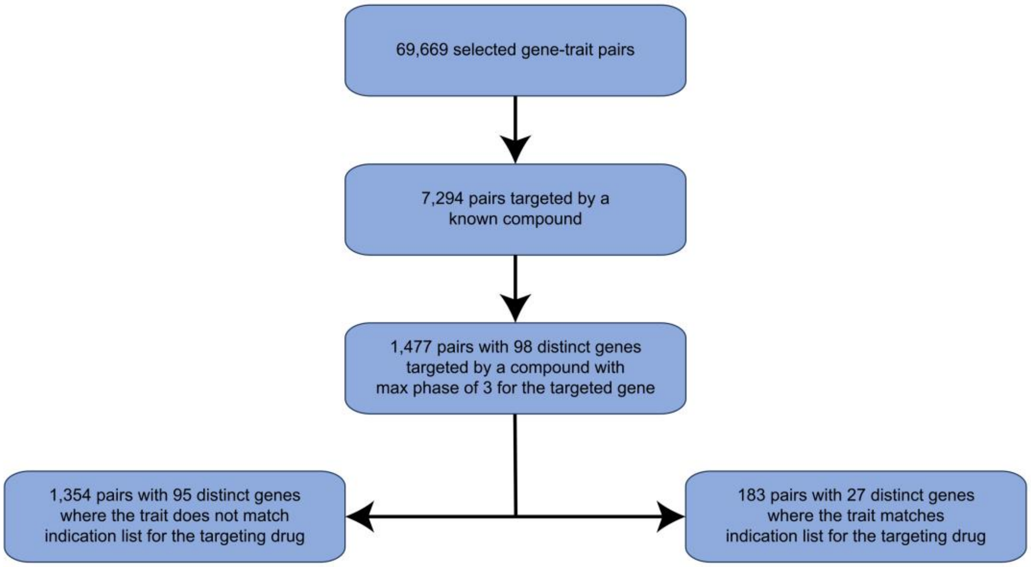

Figure S3: Flow chart showing numbers of selected gene-trait pairs that represent rediscoveries and potential repurposing opportunities.

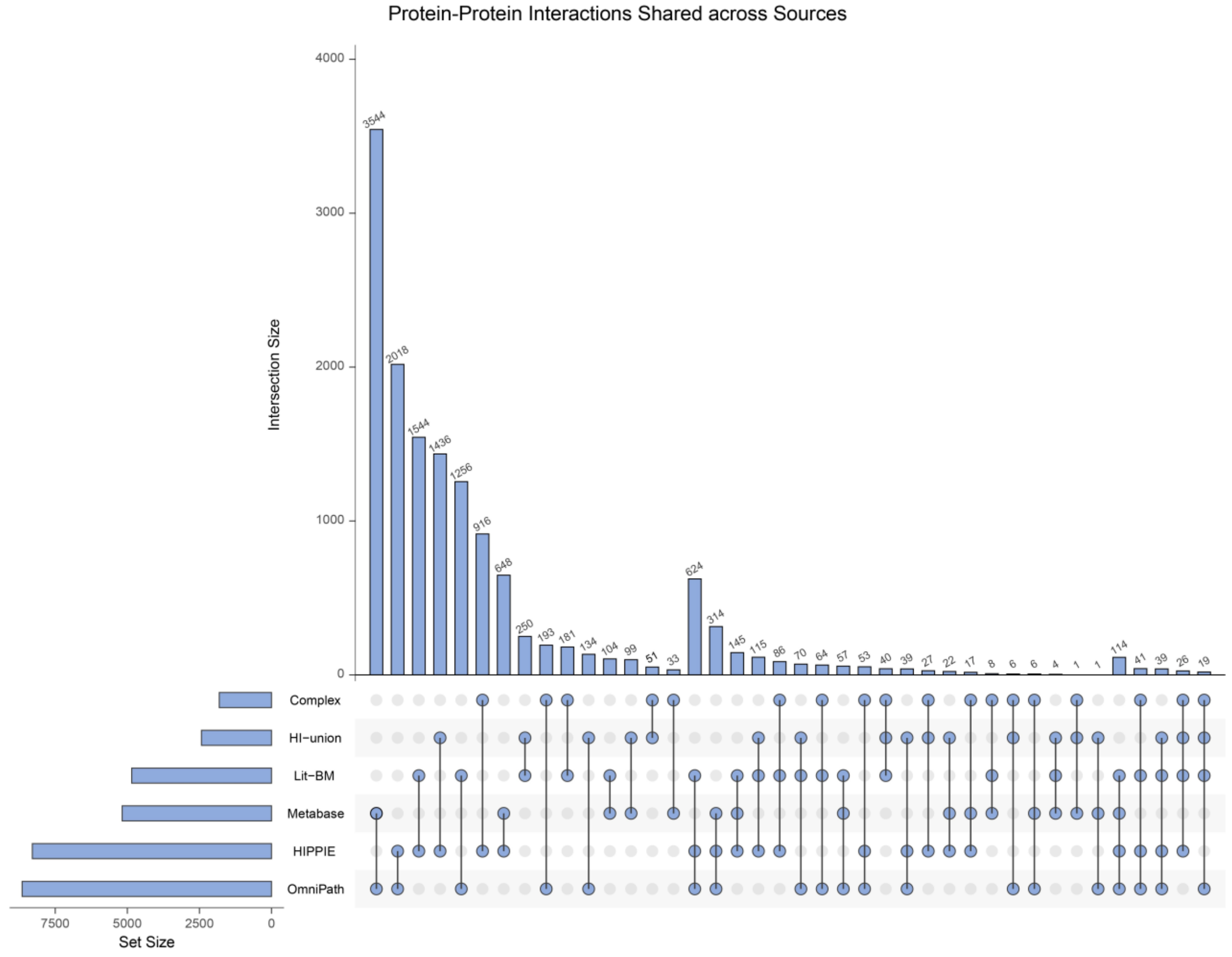

Figure S4: Upset plot depicting the intersection between the number of protein-protein pairs among the different used protein-protein interaction (PPI) datasets.

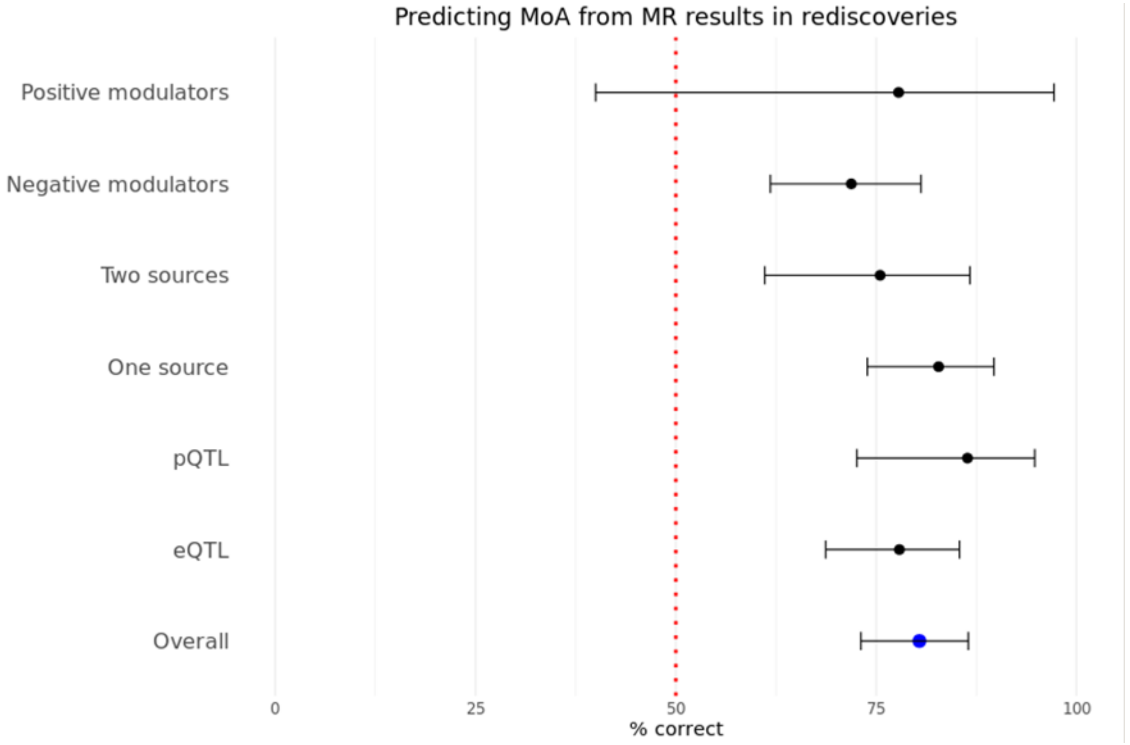

Figure S5: Prediction of correct mechanism of action (MoA) for gene-traits identified through Mendelian Randomization (MR) for approved drugs using different filters for selecting significant MR results. Positive modulators are selected MR results only targeting drugs that are positive modulators of the target; negative modulators used selected MR results only targeting drugs that are negative modulators of the target; two sources are the selected MR results identified by more than one instrument source; one source are selected MR results only identified by a single instrument source; pQTL are selected MR results identified through the use of pQTL sources; eQTL are selected MR results identified through the use of eQTL sources.

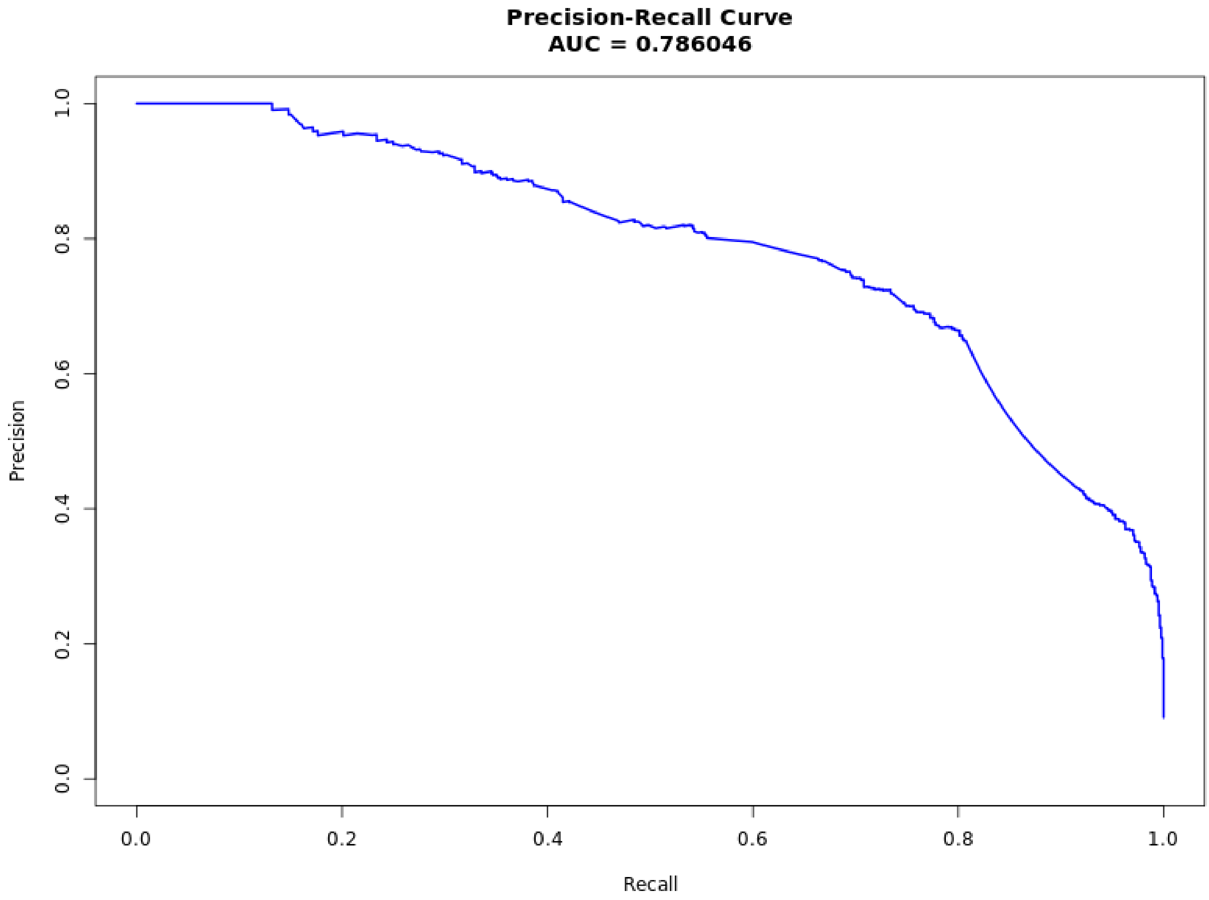

Figure S6: Precision-recall curve for our model.

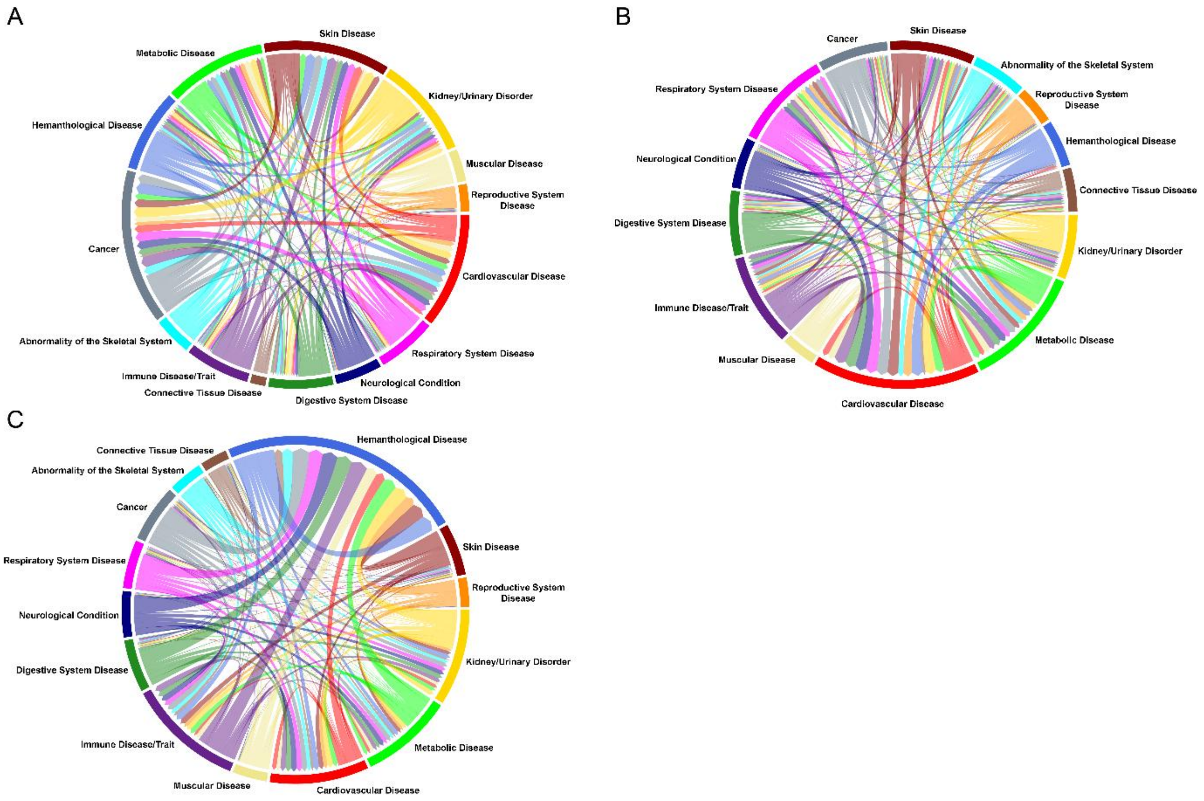

Figure S7: Chord diagrams representing instances in which significant MR gene-traits mapped to approved drugs by parent terms - excluding lab measurement traits - were found to: panel A, increase the risk of developing deleterious traits of other parent terms (suggesting a potential safety concern); panel B, decrease the risk of developing deleterious traits of other parent terms, suggesting possible drug repurposing opportunities. In panel C, a chord diagram representing all significant MR results that mapped to an approved drug, including laboratory results. Representations for all chord diagrams are from approved drug indication parent term to MR suggested drug indication/safety concern parent term.

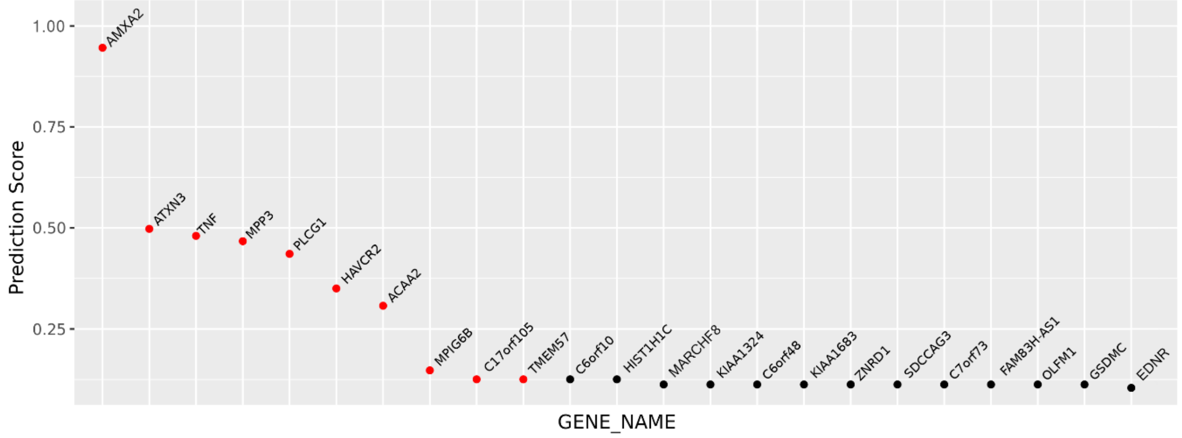

Figure S8: Top ranked genes among all genes in a significant lipid gene-trait. Predictions were calculated using the trained classifier. Red dots are gene-trait predictions with more than 70% of a positive predictive value.

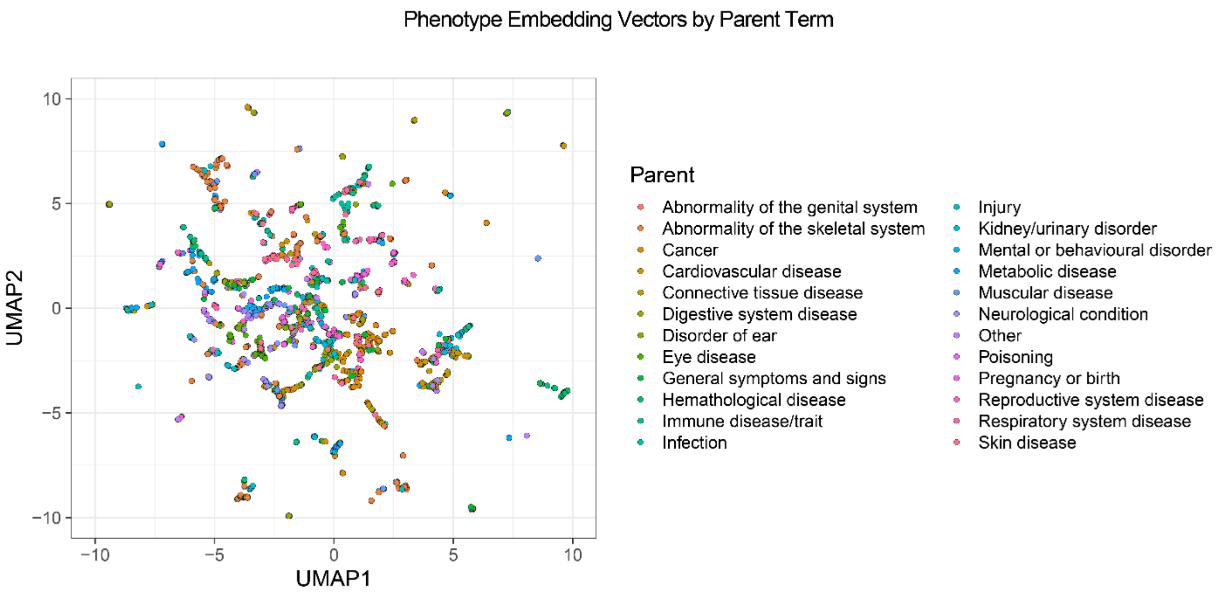

Figure S9. UMAP representation of the distance between embedding vectors used to represent the semantic status of each studied genetic phenotype. Color representation reflects assigned parent terms.

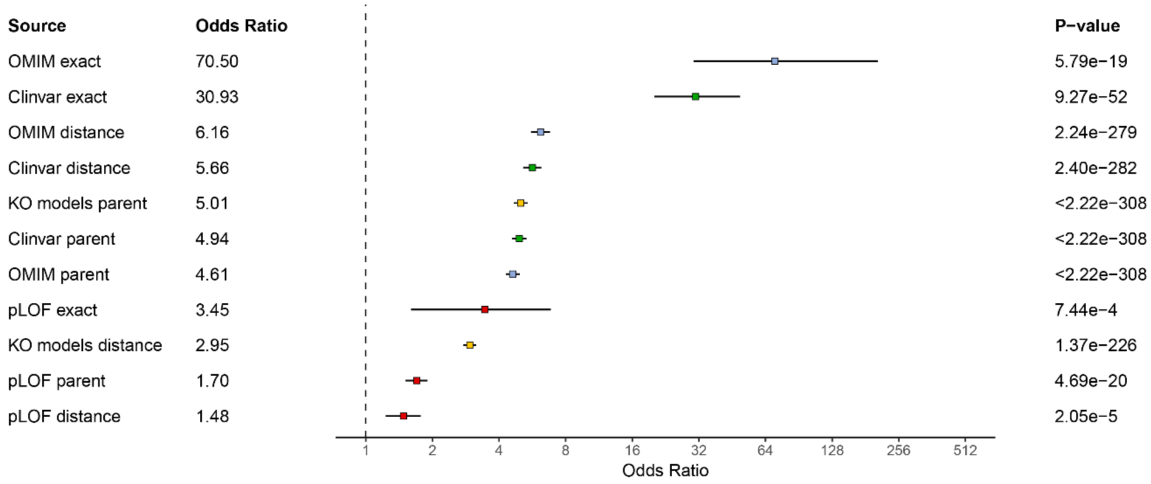

Figure S10: Forest plot of the association between the different biological features as predictors of rediscovering a relationship with an approved drug.

Supplementary Appendix 1 – Results of the harmonization between biobanks:

After all mapping efforts we have reached the number of 2011 different phenotypes. These are distributed regarding their harmonization between biobanks as:

Final Harmonization	Number of phenotypes
MVP only	327
UKBB only	265
MVP + FinnGen	30
UKBB + FinnGen	171
MVP + UKBB	770
MVP + UKBB + FinnGen	448

For all used phenotypes the distribution of the number of cases varied per biobank. Considering only phenotypes mapped to all 3 biobanks (448 phenotypes), MVP had the highest median number of cases (7608 cases, IQR 2616-21081 cases), followed by FinnGen (3350 cases, IQR 1264-10026 cases), and UKBB (1171 cases, IQR 488-3697 cases).

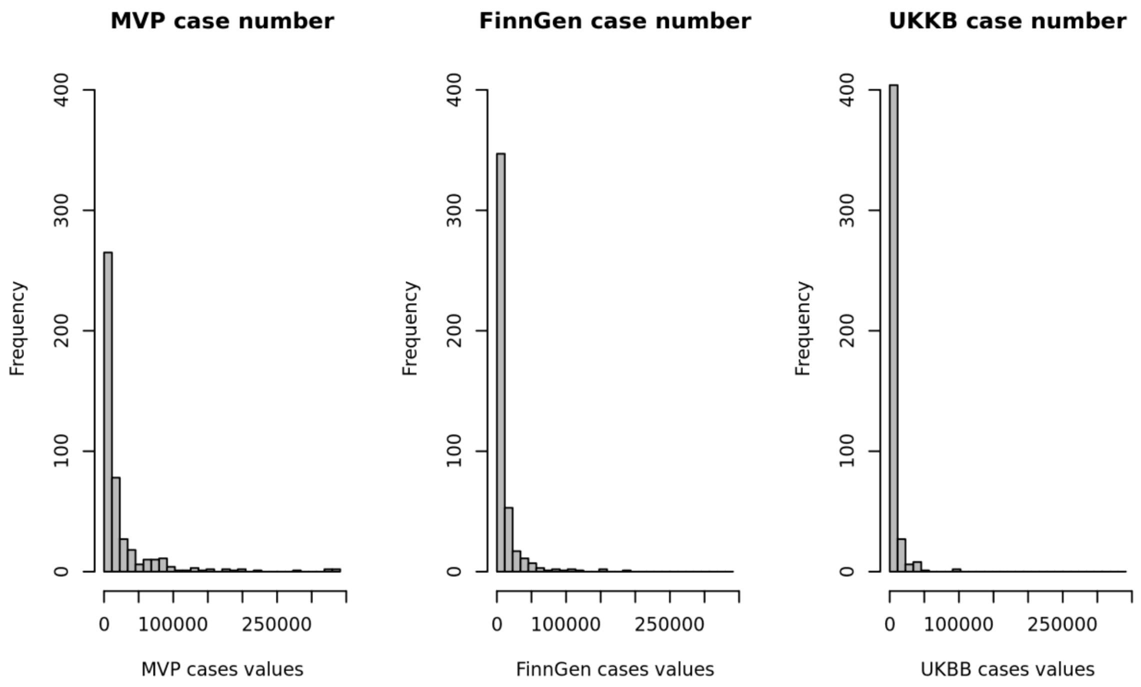

Figure 1. Histograms showing the distributions of the number of cases for different biobanks for phenotypes mapped to the 3 studied biobanks. A. cases in MVP; B. cases in FinnGen; C. Cases in UKBB. Note the trend towards phenotypes with higher number of cases in MVP, followed by FinnGen, and UKBB.

We have also explored the relative contribution of each biobank on each phenotype. Considering only phenotypes mapped to all 3 biobanks, the relative contribution of each biobank was calculated as the number of cases for that biobank divided by the total sum of cases for the 3 biobanks. The mean relative contribution for MVP was of 0.568 (sd = 0.219), for FinnGen of 0.299 (sd 0.203), and for UKBB of 0.133 (sd 0.108).

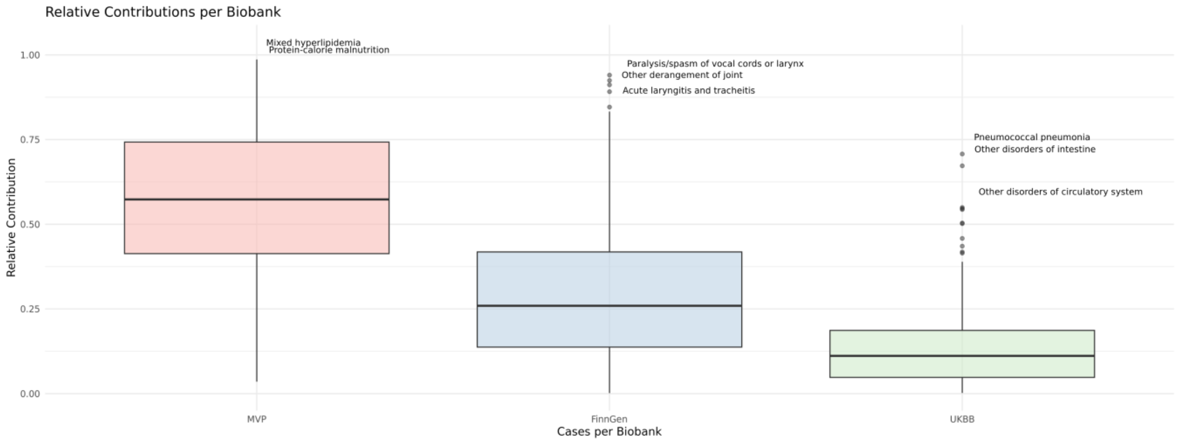

From the 448 phenotypes that were mapped between all 3 biobanks, MVP had the maximum number of cases in 329, FinnGen in 111, and UKBB in remaining 8 phenotypes.

There are several potential reasons why the relative importance of FinnGen or UKBB might be higher than MVP for specific conditions. MVP is predominantly male and several phenotypes with a higher prevalence in women were notable in the group of phenotypes where Finngen has more cases than MVP such as breast cancer, gynecomastia, and benign neoplasm of breast. Notable in this category are also autoimmune conditions such as rheumatoid arthritis, ankylosing spondylitis, and Graves disease, all with more cases in FinnGen than in MVP. Others might be more represented in FinnGen due to an explicit selection bias due to prior military service such as different types of congenital anomalies or hypertrophic cardiomyopathy, that were also more prevalent in FinnGen. Finally, it is difficult to totally exclude the possibility of different usage among medical ontological systems, leading to different prevalences of diagnosis of certain conditions due to the preferred ICD code used to describe specific diagnosis. In Supplementary Tables 1 and 2 we provide detailed numbers of the total number of cases for each phenotype as well as biobank-specific counts and biobanks relative importance for each studied phenotype.

Supplemental Appendix 2 - Descriptive Analysis of Used Genetic Instruments:

As described in the Methods section we have used genetic instruments provided by different sources and instrumented both transcript and protein levels.

The current analysis started with a total 180,967 SNP-gene combinations, 96,739 unique genetic instruments and 16,412 unique GENEs. These instruments were extracted, as detailed in methods, from 5 different sources, the eQTL data provided by GTEx V8 and eQTLGen and the pQTLs data provided by FENLAND, ARIC and DeCODE studies.

Table S1 provides the number of genetic variants for each resource used.

The higher number of instruments in GTEx V8 is explained because of the large number of tissues sampled for this dataset (49 different tissues). Analyzing only whole-blood from GTEX provides a fairer comparison between instrument resources. Taking into consideration all tissues, the number of genes

being instrumented was 16,414. From these, 11,922 were instrumented in whole-blood using at least one resource.

Table S1. *Number of unique genetic variants and genes for each resource used in this analyses.*

Resource	Type		# SNP-GENE Pairs	# Genes
GTEx V8	eQTL	All Tissues (49)	81,842	14,750
		Whole Blood	5,657	4,990
eQTLGen	eQTL		10,370	10,648
ARIC	pQTL		1,611	1,593
FENLAND	pQTL		2,880	1,509
deCODE	pQTL		4,774	1,624
**Total**		All Tissues (49)	96,739	16,412
		Whole Blood	22,806	11,920

Figure S1 considers the intersection and commonalities between resources for the number of instrumented genes.

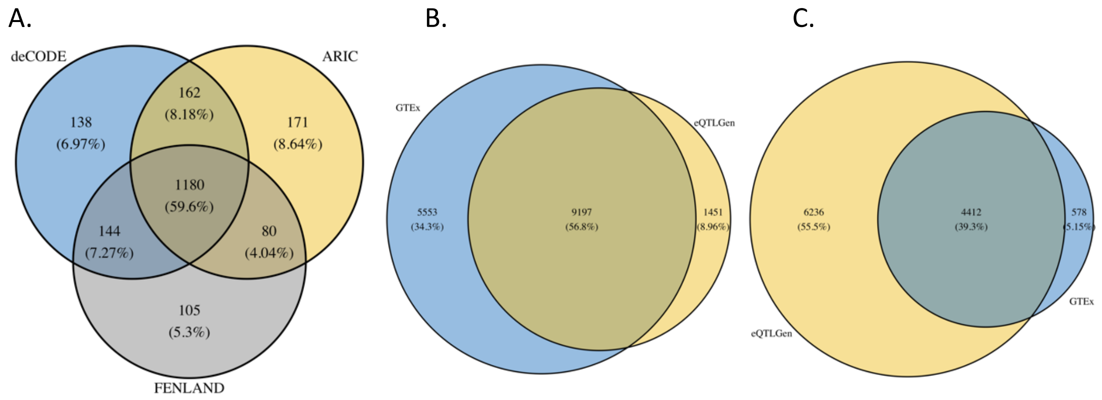

Figure S1. A. For resources targeting proteins. B for resources targeting transcripts. C. for resources targeting transcripts in Whole Blood (using GTEx Whole Blood).

There was no bias regarding resource usage. When comparing the overall distribution of instrumented genes we have. When comparing the number of genetic variants per instrumented gene we have the following distribution.

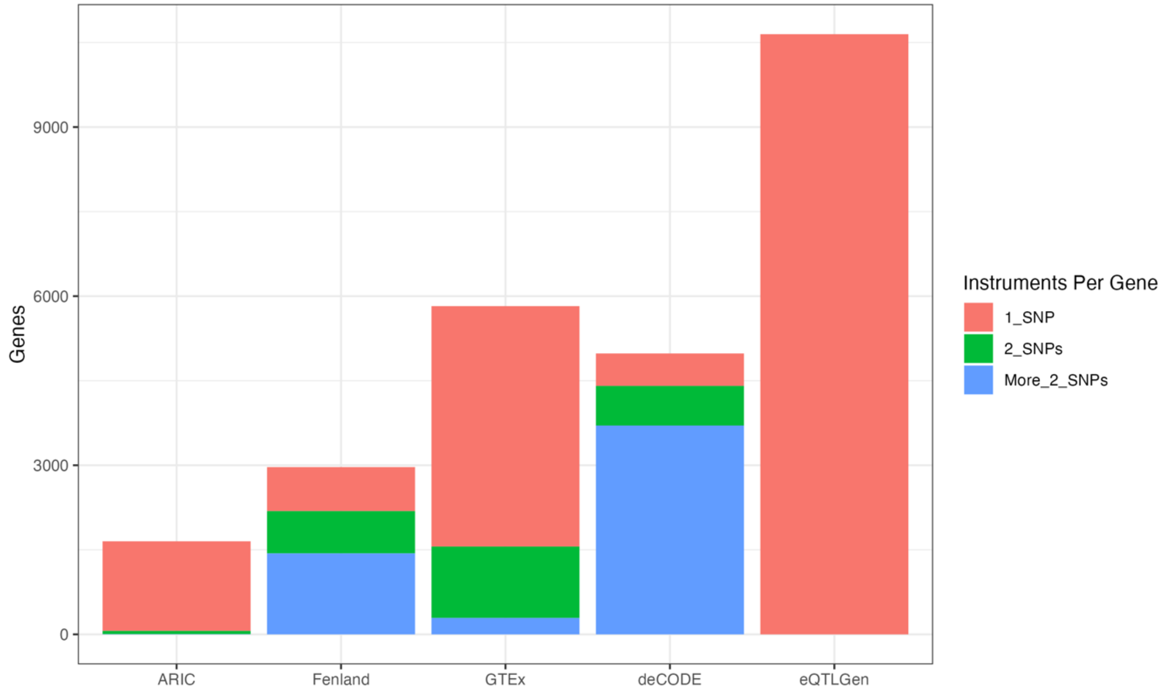

Supplementary Appendix 3 - Testing different decision rules to select QTLs for Mendelian Randomization

In deciding on how to use information from 5 different and heterogeneous (in relation to sampling, statistical power, technology and biological context) sources of genetic instruments for Mendelian Randomization we have deliberately made the compromising solution of devising a series of rules to consider a particular gene-trait as worthy of future exploration. In this sense, we only investigated gene-traits in which the MR result was significant and in which all estimated MR betas were pointing towards the same directionality. In addition, we prioritized pQTLs in favor of eQTLs and only tested the directionality of effect for pQTLs if at least one pQTL was selected as significant for a particular gene-trait. All these decisions may have impactful consequences in the overall results.

Here we provide a detailed exploration of the results when applying different selecting rules using the enrichment, sensitivity and specificity of each strategy in the evaluation task of rediscovering approved drug targets and indications. We evaluated the performance of each QTL source alone, the difference between eQTLs and pQTLs, and the impact of requiring concordance in the directionality of effect if multiple sources have significant MR results.

Individual performance of each QTL source:

GTEx

Selecting only significant results that used GTEx instruments (all tissues) we observed 451,322 significant gene-traits among all tissues distributed among 66,487 unique gene-traits. Among these gene-traits we identified 94 rediscoveries (OR 2.28, 95CI% 1.8 – 2.8, p-value = 1.03e-12). From the 66,487 unique gene-traits identified by at least one GTEx tissue, 8,580 showed discordant directionality when using

instruments derived from at least two different GTEx tissues. Excluding directionality discordant gene-traits (57,907 remaining) resulted in 90 rediscoveries and a slightly increased specificity, exemplified by an OR of 2.45 (95%CI 1.9 – 3.1, p-value = 4.48e-14).

We have also explored the sensitivity and specificity of each of the 49 different tissues available in GTEx. As expected, the number of significant gene-trait pairs was different depending on the tissue from where the instrument was derived (Figure).

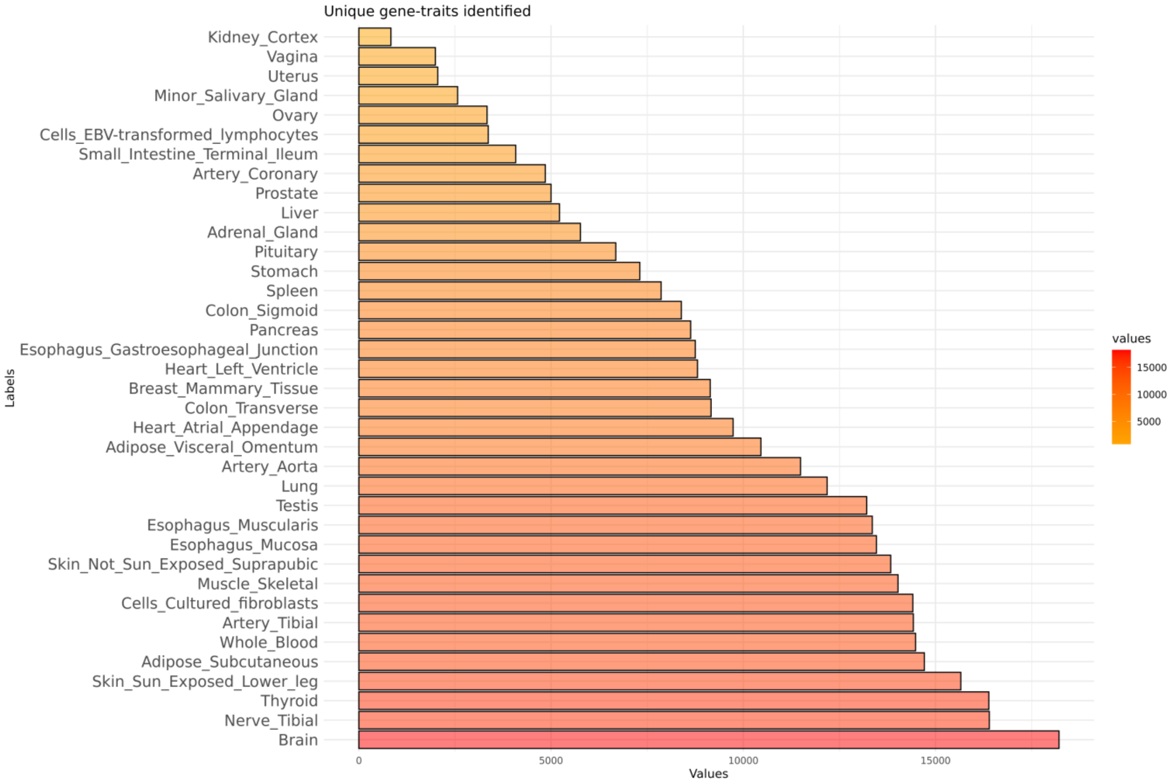

In addition, the number of rediscoveries and associated enrichment also varied amongst the different GTEx tissue instruments (Figure).

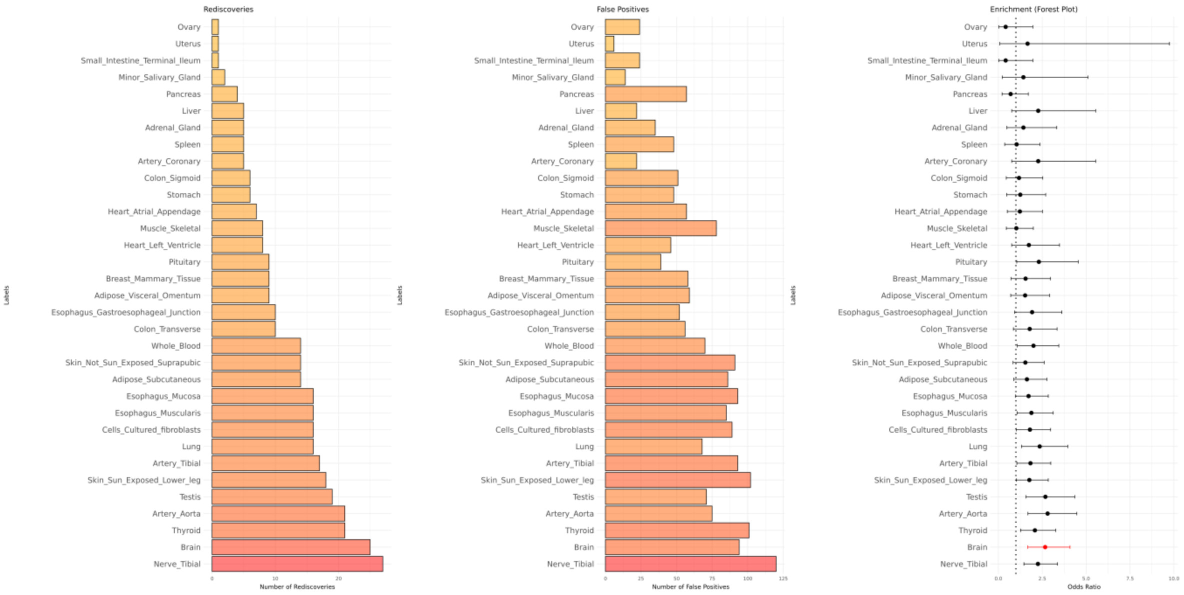

eQTLGen

Repeating the same procedure, but selecting only Mendelian Randomization results derived from eQTLGem instruments, we observed 26,576 unique gene-trait significant associations. Using only eQTLGen as an instrument resource was able to identify only 40 approved drug targets, however, we increased specificity as compared to GTEx selected gene-traits (OR 2.68, 95%CI 1.9-3.8, p-value = 3.49e-8).

Only eQTL sources:

We also tested the capacity of rediscover approved drug targets when using a combination of eQTL sources. Analyzing all significant gene-traits, regardless of directionality of effect returned 74,379 unique gene-traits. Using all results available from eQTL sources was able to identify 111 approved drug target indications. However, specificity of this approach was significantly smaller than the one observed in the main approach, with an OR of 2.40 (95%CI 1.9 – 2.9, p-value = 2.42e-16). Filtering significant results to have the same estimated directionality of effect resulted in 64,391 significant gene-traits, which led to the rediscover of 106 approved drug targets and indications. Enrichment was of 2.58 (95%CI 2.07 – 3.19), p-value <2e-16.

ARIC

Reflecting the smaller number of instrumented molecules, pQTL sources had significantly smaller sets of significant results. Using only pQTL instruments derived from ARIC we observed 4,381 significant gene-trait pairs. These identified 18 approved drug targets and indications with an enrichment of 9.50 (95%CI 4.94 – 18.18, p-value = 8.19e-12.

FENLAND

Instruments from Fenland returned 4,639 significant gene-trait pairs. These identified 27 approved drug target indications with an enrichment of 12.91 (95%CI 7.32 – 23.11, p-value = <2e-16).

DeCODE

Instruments from DeCODE returned 6,024 significant gene-trait pairs, in which there were 31 approved drug target indications. A significant gene-trait MR result using a DeCODE instrument had 10.06 (CI%95 6.09 – 16.60, p-value <2e-16) increased odds of being an approved drug target indication.

Only pQTL sources

Considering only the 3 different pQTL sources we were able to identify 8,495 significant gene-trait pairs, in which there were 36 approved drug target indications. A significant MR result identified through a pQTL instrument was associated with a 8.05 (95%CI 5.16 – 12.47, p-value <2e-16) increased odds of being an approved drug target indication. Filtering the initial 8,495 pQTL significant gene-trait pairs for only directionally concordant effects we recovered 8,355 gene-traits, in which we observed the same 36 approved drug target indications, leading to the same estimated odds of enrichment of that for the unfiltered results.

Overall eQTL sources were more sensitive and pQTL sources more specific in the task of rediscovering approved drug target indications.

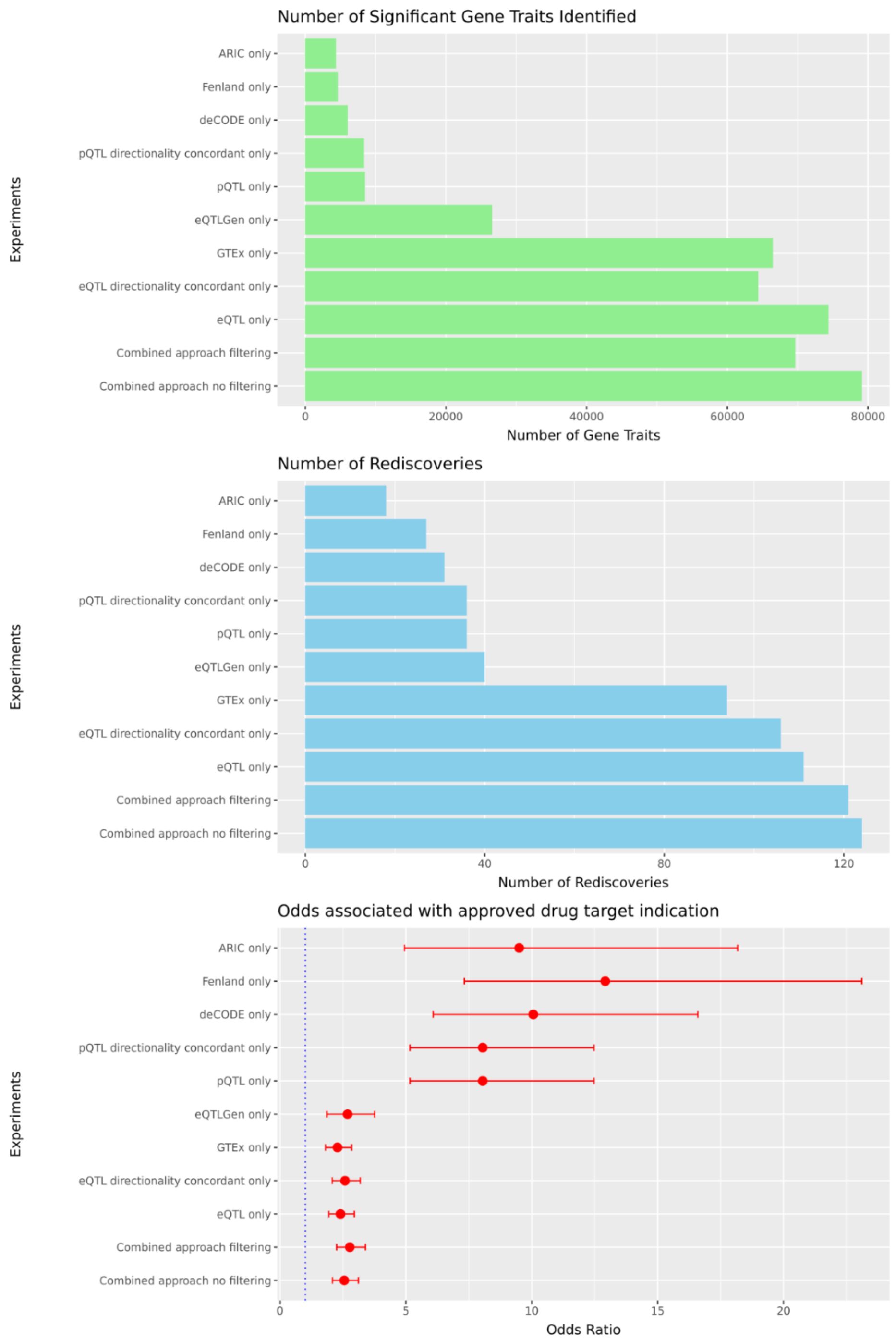

Supplementary Appendix 4 - Predictive model characteristics:

Here we describe in detail the different performance characteristics of the derived predictive model.

The overall performance, represented by the models AUC curve was very high (AUC = 0.965) (Appendix Fig 1).

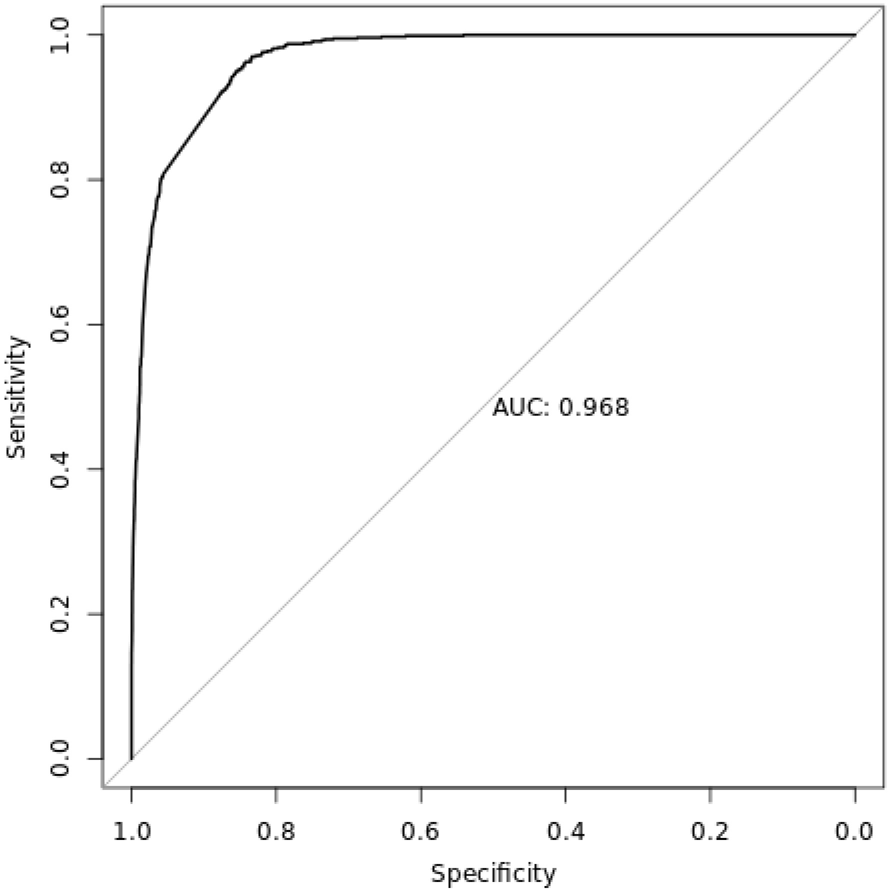

Figure 1. AUC curve of predictive model derived using testing dataset.

However, it should be noted that model performance characteristics can be very different depending on the used predicted probability threshold. In the following figure we characterize several important model performance statistics according to used probability cut-off thresholds (Appendix Figure 2).

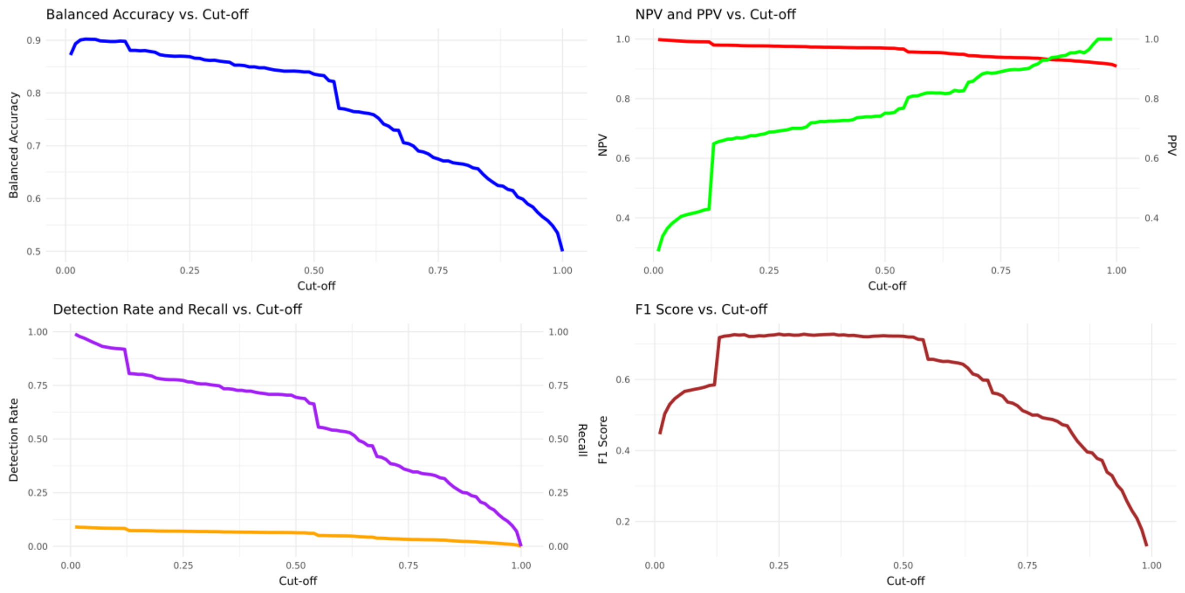

Figure 2. Model performance according to different probabilities cut-off thresholds. A. Balanced accuracy (blue line); B. NPV (negative predictive value, red line), and PPV (positive predictive value, green line); C. Detection rate (orange line) and Recall (purple line); D. F1 score (brown line).

We provide the different point estimates in Supplementary Table 10.

Due to the context of class imbalance in our model we also provide alternative model performance descriptors.

Precision-recall AUC was 0.79 (Figure 3).

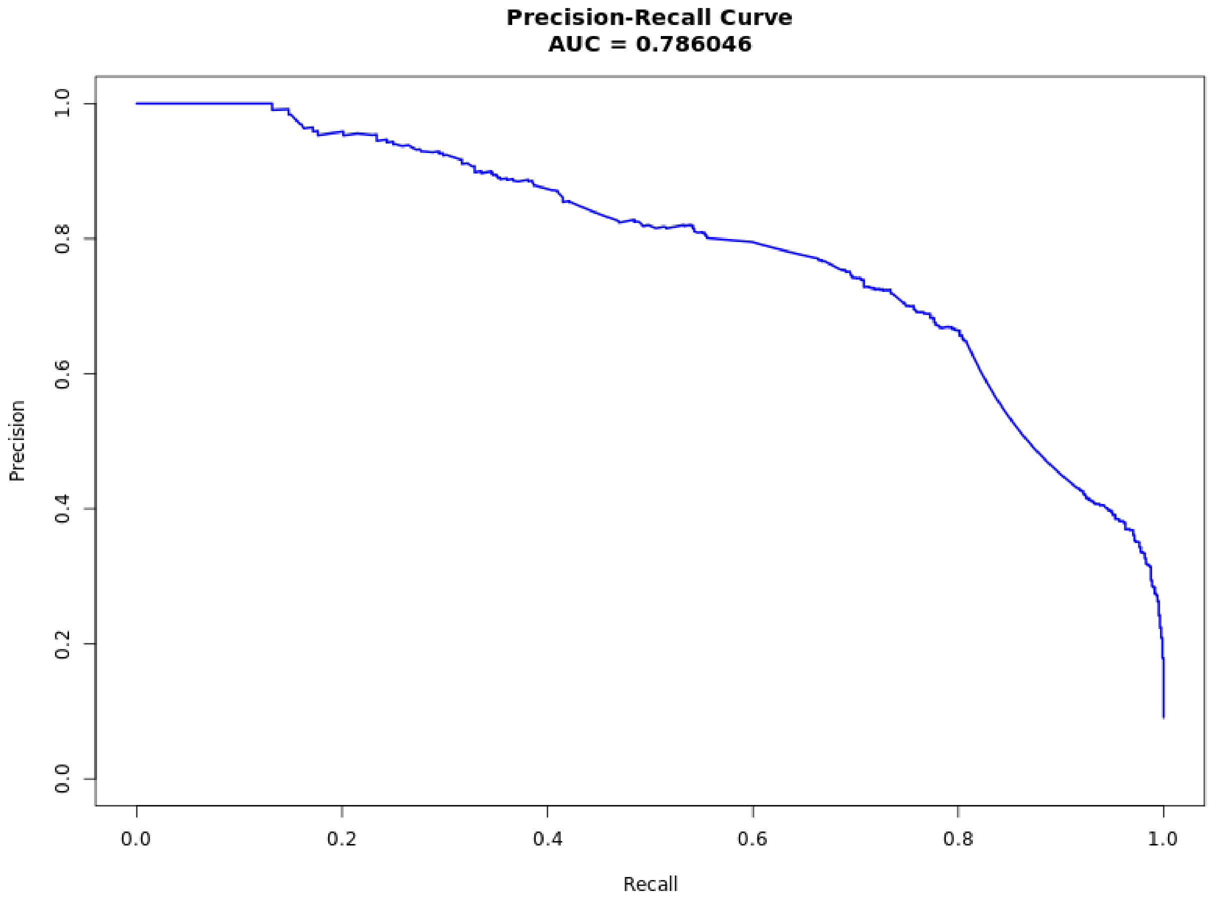

Figure 3. Precision-recall curve using results from testing set.

From a decision perspective, Lift and Gain plots suggest that using a threshold of predicted probability equal or above 10% may capture most gains elicited by the model without contaminating the sample of selected results with a significant number of false-positives (Figure 4). However, it is important to point out that even predicted probabilities above 1% have significant enrichment for approved drug targets and indications.

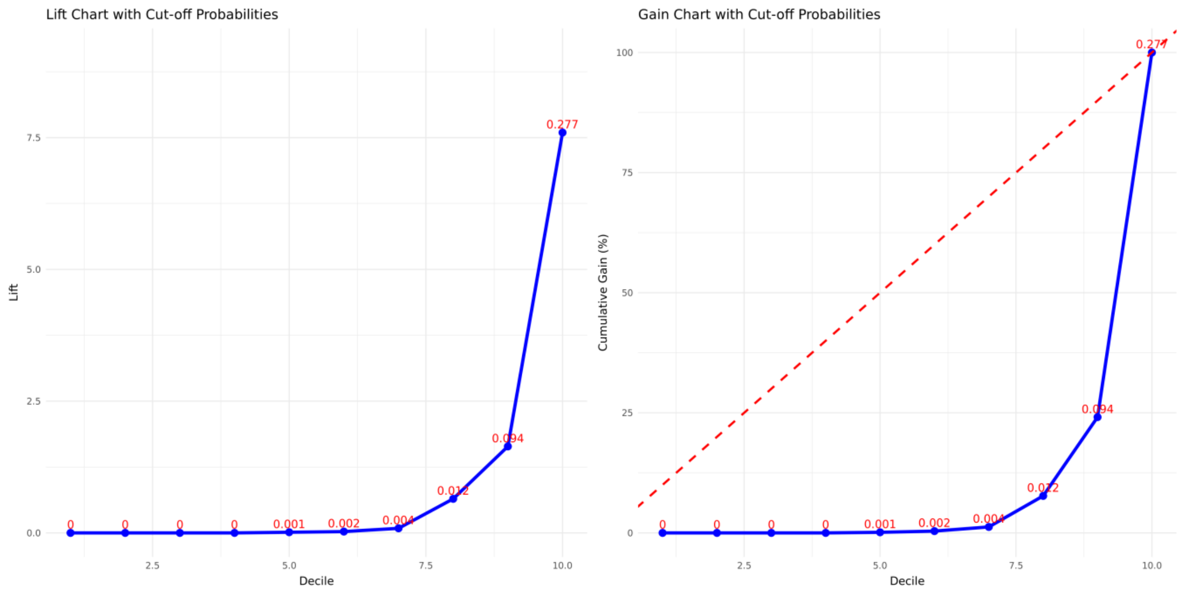

Figure 4. Lift (left) and gain (right) charts derived from testing dataset. Within each decile we plot the observed cut-off predicted probability.

Supplementary Appendix 5 - Comparing a Mendelian Randomization approach to other propositions to map associated gene to genetic associated signals for drug discovery target identification

Using the identification of approved drug targets and approved indications (our rediscovery concept) we have benchmarked 3 different approaches to map genes from genetic associated variants: the Variant Effect Prediction (VEP) from Ensemble, V2G from OpenTargets, and a naïve nearest gene mapping approach. For all these comparisons we have used a similar approach as the used in our main MR approach to define the p-value cut-off. For selecting associated genetic variants to map to genes, we have defined the p-value cut-off as 5 x 10^{-8} divided by the number of GWAS traits (5e-8/2003 = 2.5e-11).

Nearest Gene

Arguably the most naïve approach is to map each significant GWAS variant to its nearest gene. We have carried out this procedure and identified 601,343 unique gene-trait pairs (53,997 unique genes, including lncRNA genes and mapped pseudogenes and 1730 unique phenotypes). Using the simple nearest gene approach was able to rediscover 259 approved drug target indications (163 unique drug targets). The increased number of rediscoveries, as compared to a Mendelian Randomization approach, comes at the cost of reduced specificity and the overall enrichment of approved drug target in significant gene-traits defined by the nearest gene was of 1.79 (1.6 – 2.1), p-value <2e-16.

Variant Effect Prediction strategy

We mapped all variants that reached the pre-specified p-value cut-off to genes using VEP. For all significant phenotype-variant associations, unique variants were arranged in sequential order and then converted into Variant Call Format (VCF). This list of variants was then partitioned into 18 files to adhere

to the 50MB file size maximum for query submissions to the Ensembl Variant Effect Predictor (VEP) web tool (McLaren et al., 2016). These files were then submitted as queries to the VEP web tool using the Human GRCh38.p14 assembly and the Ensembl/GENCODE gene set to predict the associated genes and molecular consequence type for each query variant (McLaren et al., 2016; Hunt et al., 2022). The resulting variant-gene pairs were downloaded in text (TXT) file format from the VEP web tool and concatenated in R. These variant-gene pairs were then mapped back to the original phenotype-variant pairings using chromosome and starting base position to connect the two datasets and produce a full list of phenotype-gene associations.

This resulted in 105,864 unique gene-traits (1,340 unique phenotypes and 12,754 mapped genes). VEP derived gene-traits were able to rediscover 107 of approved drug target indications (67 unique drug targets). The overall enrichment for a gene-trait identified through VEP was of 2.6 (2.1 – 3.2), p-value <2e-16.

V2G strategy

Using V2G we mapped all significant GWAS variants to a gene. The latest set of scored human genome variant-to-gene pairings from Open Target Genetics was downloaded from their File Transfer Protocol (FTP) database (Ghoussaini et al., 2021). The file set was concatenated into a single file in R, and then mapped to the full list of unique variants from the complete set of significant phenotype-variant associations using chromosome and starting base position to represent unique variants. The resulting list of variant-gene pairs was then mapped back to the original phenotype-variant pairings, again using chromosome and starting base position to connect the two datasets and produce a full list of phenotype-gene associations. Finally, gene Ensembl IDs were mapped to their corresponding HUGO Gene Nomenclature Committee (HGNC) gene symbols, using REST API queries of the Ensembl gene database (Yates et al., 2014).

From all significantly associated markers, V2G mapped 11,872 genes to 1,340 unique phenotypes, resulting in 122,558 unique gene-trait pairs. V2G derived gene-traits rediscovered 151 approved drug target indications. The overall enrichment for a gene-trait identified through V2G was similar to that of VEP and smaller than the identified by Mendelian Randomization using the pre-defined p-value cut-off (2.51, 95%CI 2.1 – 3.0, p-value <2e-16).

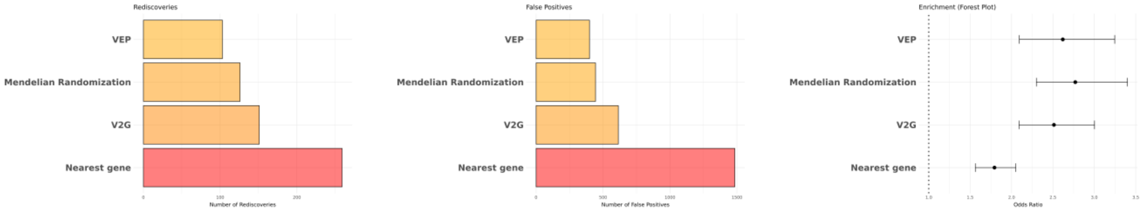

McLaren, W., Gil, L., Hunt, S. E., Riat, H. S., Ritchie, G. R., Thormann, A., Flicek, P., & Cunningham, F. (2016). The Ensembl Variant Effect Predictor. Genome biology, 17(1), 122. https://doi.org/10.1186/s13059-016-0974-4

Hunt, S. E., Moore, B., Amode, R. M., Armean, I. M., Lemos, D., Mushtaq, A., Parton, A., Schuilenburg, H., Szpak, M., Thormann, A., Perry, E., Trevanion, S. J., Flicek, P., Yates, A. D., & Cunningham, F. (2022).

Annotating and prioritizing genomic variants using the Ensembl Variant Effect Predictor-A tutorial. Human mutation, 43(8), 986–997. https://doi.org/10.1002/humu.24298

Ghoussaini, M., Mountjoy, E., Carmona, M., Peat, G., Schmidt, E. M., Hercules, A., Fumis, L., Miranda, A., Carvalho-Silva, D., Buniello, A., Burdett, T., Hayhurst, J., Baker, J., Ferrer, J., Gonzalez-Uriarte, A., Jupp, S., Karim, M. A., Koscielny, G., Machlitt-Northen, S., Malangone, C., … Dunham, I. (2021). Open Targets Genetics: systematic identification of trait-associated genes using large-scale genetics and functional genomics. Nucleic acids research, 49(D1), D1311–D1320. https://doi.org/10.1093/nar/gkaa840

Yates, A., Beal, K., Keenan, S., McLaren, W., Pignatelli, M., Ritchie, G. R., Ruffier, M., Taylor, K., Vullo, A., & Flicek, P. (2014). The ENSEMBL REST API: Ensembl data for any language. Bioinformatics, 31(1), 143–145. https://doi.org/10.1093/bioinformatics/btu613

Supplementary File 1 – Significant gene – trait pa

Supplementary File 3 - VA Million Veteran Program Core Acknowledgements for Publications June 2025

MVP Program Office

- Sumitra Muralidhar, Ph.D., Program Director

US Department of Veterans Affairs, 810 Vermont Avenue NW, Washington, DC 20420

- Jennifer Moser, Ph.D., Associate Director, Scientific Programs

US Department of Veterans Affairs, 810 Vermont Avenue NW, Washington, DC 20420

- Jennifer E. Deen, B.S., Associate Director, Cohort & Public Relations

US Department of Veterans Affairs, 810 Vermont Avenue NW, Washington, DC 20420

MVP Executive Committee

- Co-Chair: Philip S. Tsao, Ph.D.

VA Palo Alto Health Care System, 3801 Miranda Avenue, Palo Alto, CA 94304

- Co-Chair: Sumitra Muralidhar, Ph.D.

US Department of Veterans Affairs, 810 Vermont Avenue NW, Washington, DC 20420

- J. Michael Gaziano, M.D., M.P.H.

VA Boston Healthcare System, 150 S. Huntington Avenue, Boston, MA 02130

- Elizabeth Hauser, Ph.D.

Durham VA Medical Center, 508 Fulton Street, Durham, NC 27705

- Amy Kilbourne, Ph.D., M.P.H.

VA HSR&D, 2215 Fuller Road, Ann Arbor, MI 48105

- Michael Matheny, M.D., M.S., M.P.H.

VA Tennessee Valley Healthcare System, 1310 24th Ave. South, Nashville, TN 37212

- Dave Oslin, M.D.

Philadelphia VA Medical Center, 3900 Woodland Avenue, Philadelphia, PA 19104

- Deepak Voora, MD

Durham VA Medical Center, 508 Fulton Street, Durham, NC 27705

MVP Co-Principal Investigators

- J. Michael Gaziano, M.D., M.P.H.

VA Boston Healthcare System, 150 S. Huntington Avenue, Boston, MA 02130

- Philip S. Tsao, Ph.D.

VA Palo Alto Health Care System, 3801 Miranda Avenue, Palo Alto, CA 94304

MVP Core Operations

- Jessica V. Brewer, M.P.H., Director, MVP Cohort Operations

VA Boston Healthcare System, 150 S. Huntington Avenue, Boston, MA 02130

- Mary T. Brophy M.D., M.P.H., Director, VA Central Biorepository

VA Boston Healthcare System, 150 S. Huntington Avenue, Boston, MA 02130

- Kelly Cho, M.P.H, Ph.D., Director, MVP Phenomics

MVP Core Acknowledgements for Publications_June 2025

VA Boston Healthcare System, 150 S. Huntington Avenue, Boston, MA 02130

- Lori Churby, B.S., Director, MVP Regulatory Affairs

VA Palo Alto Health Care System, 3801 Miranda Avenue, Palo Alto, CA 94304

- Scott L. DuVall, Ph.D., Director, VA Informatics and Computing Infrastructure (VINCI)

VA Salt Lake City Health Care System, 500 Foothill Drive, Salt Lake City, UT 84148

- Saiju Pyarajan Ph.D., Director, Data and Computational Sciences

VA Boston Healthcare System, 150 S. Huntington Avenue, Boston, MA 02130

- Robert Ringer, Pharm.D., Director, VA Albuquerque Central Biorepository

New Mexico VA Health Care System, 1501 San Pedro Drive SE, Albuquerque, NM 87108

- Luis E. Selva, Ph.D., Director, MVP Biorepository Coordination

VA Boston Healthcare System, 150 S. Huntington Avenue, Boston, MA 02130

- Shahpoor (Alex) Shayan, M.S., Director, MVP PRE Informatics

VA Boston Healthcare System, 150 S. Huntington Avenue, Boston, MA 02130

- Brady Stephens, M.S., Principal Investigator, MVP Information Center

Canandaigua VA Medical Center, 400 Fort Hill Avenue, Canandaigua, NY 14424

- Stacey B. Whitbourne, Ph.D., Director, MVP Cohort Development and Management

VA Boston Healthcare System, 150 S. Huntington Avenue, Boston, MA 0213
